# The travel speeds of large animals are limited by their heat-dissipation capacities

**DOI:** 10.1371/journal.pbio.3001820

**Published:** 2023-04-18

**Authors:** Alexander Dyer, Ulrich Brose, Emilio Berti, Benjamin Rosenbaum, Myriam R. Hirt

**Affiliations:** 1 EcoNetLab, German Centre for Integrative Biodiversity Research Halle-Jena-Leipzig, Leipzig, Germany; 2 Institute of Biodiversity, Friedrich-Schiller-University Jena, Jena, Germany; Lund University, SWEDEN

## Abstract

Movement is critical to animal survival and, thus, biodiversity in fragmented landscapes. Increasing fragmentation in the Anthropocene necessitates predictions about the movement capacities of the multitude of species that inhabit natural ecosystems. This requires mechanistic, trait-based animal locomotion models, which are sufficiently general as well as biologically realistic. While larger animals should generally be able to travel greater distances, reported trends in their maximum speeds across a range of body sizes suggest limited movement capacities among the largest species. Here, we show that this also applies to travel speeds and that this arises because of their limited heat-dissipation capacities. We derive a model considering how fundamental biophysical constraints of animal body mass associated with energy utilisation (i.e., larger animals have a lower metabolic energy cost of locomotion) and heat-dissipation (i.e., larger animals require more time to dissipate metabolic heat) limit aerobic travel speeds. Using an extensive empirical dataset of animal travel speeds (532 species), we show that this *allometric heat-dissipation model* best captures the hump-shaped trends in travel speed with body mass for flying, running, and swimming animals. This implies that the inability to dissipate metabolic heat leads to the saturation and eventual decrease in travel speed with increasing body mass as larger animals must reduce their realised travel speeds in order to avoid hyperthermia during extended locomotion bouts. As a result, the highest travel speeds are achieved by animals of intermediate body mass, suggesting that the largest species are more limited in their movement capacities than previously anticipated. Consequently, we provide a mechanistic understanding of animal travel speed that can be generalised across species, even when the details of an individual species’ biology are unknown, to facilitate more realistic predictions of biodiversity dynamics in fragmented landscapes.

## Abstract 2

Reported trends in the maximum travel speeds of animals across a range of body sizes suggest that the largest species have limited movement capacities. By simultaneously considering body mass-dependent constraints on energy utilization and heat dissipation, this study provides a mechanistic explanation of animals’ cruising speeds.

## Introduction

The movements of animals over land, in water, and through air are a central component of several ecological processes that together give rise to patterns of biodiversity and ecosystem functioning [1–3]. Movement behaviours such as foraging, dispersal, and migration grant animals access to resources and reproductive opportunities and are, therefore, critical to their long-term survival within spatially fragmented ecosystems. However, despite these potential benefits, movement behaviours usually carry with them considerable costs. For example, during transience—the phase of dispersal, which enables large-scale displacements [4,5]—animals must contend with extended bouts of elevated metabolic expenditure in order to sustain locomotion through a, typically, hostile landscape (reviewed in [6]). Such trade-offs contribute to the emergence of complex spatial dynamics within landscapes that are crucial to species persistence [7–9]. In light of mounting evidence suggesting that anthropogenic perturbations such as landscape fragmentation, climate change, and land-use change disrupt the natural movements of animals [10–14], a general mechanistic understanding of animals’ movement capacities represents a critical step towards addressing the consequences of landscape connectivity for patterns of biological diversity [15–18].

A central component of an animal’s movement capacity is its sustained (i.e., aerobic) travel speed, which fundamentally depends on its locomotion mode, body mass, and the temperature that it experiences. Unlike the travel speeds of foraging animals, which can be predicted by optimal foraging theory, the travel speeds of nonforaging animals are subject to context-dependent trade-offs between energy costs, travel time, and distance [19,20]. Among animals of similar size, flying is generally faster than running and swimming, while within each locomotion mode, larger animals tend to move faster and farther than smaller animals ([21–23]; but see [24,25]). Although allometric relationships have achieved generality in relating animal body mass to the mechanical- [26–29] and metabolic energy costs of locomotion [30–33], devising a general allometric model that can predict the speeds that animals are capable of sustaining has remained a challenge: Among existing models, which, typically, describe a power-law scaling relationship between travel speed and animal body mass (but see [34]), there are significant discrepancies in the reported values of the allometric scaling exponent across disparate groups of flying, running, and swimming animals (e.g., [22,35–39]). This suggests that models based solely on the mechanical and metabolic energy demands of locomotion are insufficient to predict the travel speeds of animals across a sufficiently wide range of taxonomic groups and locomotion modes.

Based on prior models of maximum speed [40,41], we also consider a hump-shaped relationship between travel speed and body mass. So far, very few studies have explicitly considered the importance of metabolic heat—an inevitable by-product of muscular contractions—in limiting the speeds that animals can travel at during extended locomotion bouts (but see [42–44]). In order for an animal’s core body temperature to remain stable and within its thermal limits, it is essential that the heat that its body dissipates to the ambient environment is sufficient to balance the excess heat that its muscles produce during locomotion. If heat-dissipation cannot offset metabolic heat production, animals must decrease their metabolic demands and, thus, their speed in order to avoid hyperthermia. Consequently, we argue that a general mechanistic model that can predict the travel speeds of animals must account for both the fate of energy that goes towards the performance of useful work as well as the fate of energy that is dissipated internally as heat.

We derive a general allometric model that considers how fundamental biophysical constraints associated with the supply, utilisation, and dissipation of energy and heat limit the travel speeds of flying, running, and swimming animals during extended locomotion bouts. Our model builds on previous biomechanical and metabolic approaches by considering the body mass dependence of (1) aerobic metabolism [45,46] and (2) the metabolic cost of locomotion [30,31,33]. Unlike previous models, we also consider how (3) animals’ capacity to dissipate metabolic heat fundamentally constrains their capacity for sustained locomotion. This ultimately leads to the prediction of a hump-shaped relationship between travel speed and body mass. We established an exhaustive dataset on empirical animal travel speeds to test (1) whether this new heat-dissipation model provides more accurate predictions of animal travel speeds than conventional power-law models and (2) if it makes consistent predictions across locomotion modes and ecosystem types. Ultimately, our approach provides a mechanistic model of animal travel speed that can be generalised across species, even when the details of an individual species’ biology are unknown.

## Model development

**Fig 1:**
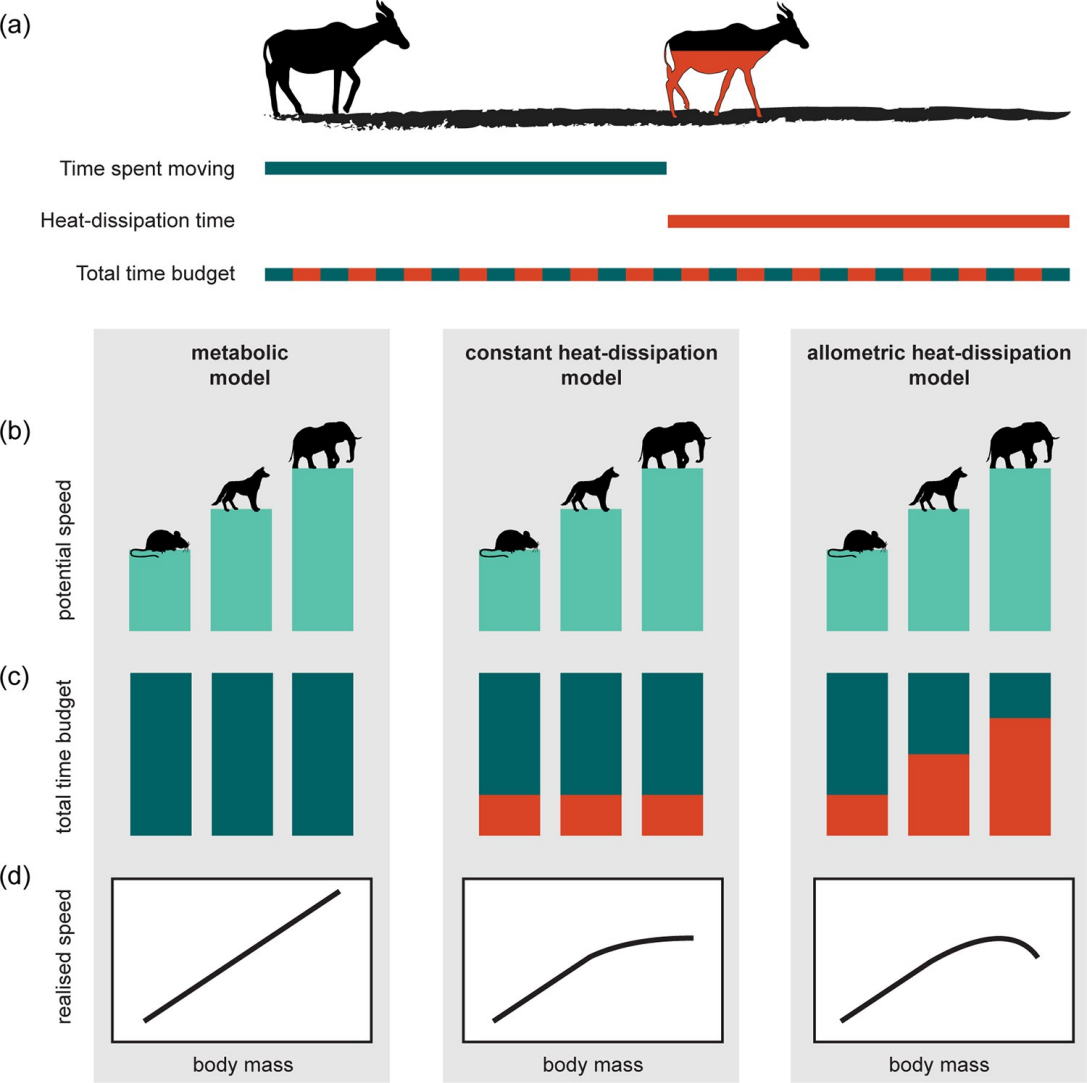
Concept of heat-dissipation time as a fundamental constraint to the realised travel speeds of animals. (**a**) Travel speed represents the distance moved divided by the total time budget allocated towards a sustained movement behaviour (e.g., exploration, dispersal, migration). The time spent moving a unit distance corresponds to the production of metabolic heat by contracting muscles as they perform the mechanical work required for locomotion. In order for the core body temperature to remain stable, a fraction of the total time budget is allocated towards heat-dissipation to offset the heat that is produced during locomotion; this takes place cyclically at small time-steps (e.g., between each stride). An increase in heat-dissipation time, therefore, corresponds to a reduced stride frequency and a net decrease in the realised travel speed. (**b**) A greater supply of metabolic power combined with a higher locomotion efficiency allows larger animals to sustain higher potential travel speeds. (**c**) The fraction of the total time budget, relative to body mass, that is allocated towards locomotion (blue) or heat-dissipation (red): (1) time is exclusively allocated towards locomotion (*metabolic model*); (2) all species allocate a constant (i.e., body mass–independent) fraction towards heat-dissipation (*constant heat-dissipation model*); (3) larger animals allocate a larger fraction towards heat-dissipation (*allometric heat-dissipation model*). (**d**) The allocation of heat-dissipation time determines the realised travel speed that can be sustained, yielding a (1) power-law (*metabolic model*), (2) saturating (*constant heat-dissipation model*), or (3) hump-shaped (*allometric heat-dissipation model*) allometric scaling model.

We derive 3 alternative models of how animal travel speed scales with body mass. The models are based on varying assumptions of how, for a given distance moved, the total time budget during extended locomotion bouts is split into time spent moving and heat-dissipation time (Fig 1A). For simplicity, we retain the concept of discrete time budgets for locomotion and heat-dissipation, while empirically both can take place at infinitely small time-steps (e.g., between each stride) without violating the assumptions of the concept (Fig 1A, lowest bar). In other words, animals do not need to stop to dissipate heat; instead, they continuously allocate part of their total time budget towards heat-dissipation by moving more slowly. The 3 models are based on the same allometric relationships for metabolic power generation and locomotion efficiency and, therefore, predict the same potential travel speed (Fig 1B). However, they differ in whether they assume that heat-dissipation time is (1) not necessary (*metabolic model*), (2) constant across all species (*constant heat-dissipation model*), or (3) increases with body mass (*allometric heat-dissipation model*, Fig 1C). Consequently, they predict that the realised travel speed scales with body mass as a power law (*metabolic model*), a saturating function (*constant heat-dissipation model*), or a hump-shaped function (*allometric heat-dissipation model*, Fig 1D). In the following, we provide an overview of the model derivation (see also Table 1), while the detailed derivation is provided as a supporting information (see S1 Text).

**Table 1:** Overview of 3 alternative allometric locomotion models and their corresponding mechanistic hypotheses.

	Metabolic model	Constant heat-dissipation model	Allometric heat-dissipation model
Step 1: Travel speed	$v=\frac{D}{t_{total}}$		
Step 2: Distance moved	*D* = *P*_*i*_ · *E*_*max*_ · *t*_*move*_		
Step 3: Metabolic power input	*P*_*i*_ = *P*_0_ · *M*^*a*^		
Step 4: Maximum locomotion efficiency	*E*_*max*_ = *E*_0_ · *M*^*b*^		
Step 5: Potential travel speed	$v=\frac{(P_{0}{\cdot E}_{0})\cdot M^{a+b}\cdot t_{move}}{t_{total}}=\frac{v_{0}\cdot M^{c}\cdot t_{move}}{t_{total}}$		
Step 6: Total time budget for travel	*t*_*total*_ = *t*_*move*_	*t*_*total*_ = *t*_*move*_ + *t*_*diss*_	
Step 7: Scaling of heat-dissipation time	*t*_*diss*_ = 0	*t*_*diss*_ = *k*_0_ · *D*	*t*_*diss*_ = *k*_*λ*_ · *M*^*d*^ · *D*
Step 8: Full models	*v* = *v*_0_ · *M*^*c*^	$v=\frac{\frac{1}{k_{0}}\cdot M^{c}}{M^{c}+\frac{1}{v_{0}\cdot k_{0}}}$	$v=\frac{\frac{1}{k_{\lambda }}\cdot M^{c}}{M^{c+d}+\frac{1}{v_{0}\cdot k_{\lambda }}}$

Description of model parameters: travel speed, *v* (*m s*^−1^); distance moved, *D* (*m*); normalisation constants for metabolic power input, *P*_0_ (*J s*^−1^ *kg*^−*a*^), and maximum locomotion efficiency, *E*_0_ (*m J*^−1^ *kg*^−*b*^); locomotion rate constant that varies among locomotion modes, *v*_0_ (*m s*^−1^ *kg*^−*c*^); animal body mass, *M* (*kg*); total time budget allocated towards a sustained movement behaviour, *t*_*total*_ (s); time spent moving, *t*_*move*_ (s); heat-dissipation time, *t*_*diss*_ (s); heat-dissipation time constants, *k*_0_ (*s m*^−1^) and *k*_*λ*_ (*s m*^−1^ *kg*^−*d*^); scaling exponents describing the body mass dependence of metabolic power input, *a*, maximum locomotion efficiency, *b*, potential travel speed, *c*, and heat-dissipation time, *d*.

All 3 allometric models of travel speed have the first 5 steps of model derivation in common: First, travel speed is equal to the distance moved divided by the total travel time (Table 1, step 1). Second, distance moved is predicted by the product of whole-organism metabolic power input and locomotion efficiency (Table 1, step 2). Third, metabolic power input scales with body mass as a power law (Table 1, step 3). Fourth, the maximum locomotion efficiency, the reciprocal of the minimum absolute metabolic cost of locomotion, also follows a power-law scaling relationship with body mass (Table 1, step 4). Taken together, these terms produce an allometric model of potential travel speed that is shared by all 3 models (Table 1, step 5, Fig 1B). The 3 models differ in their assumptions on total time budgets (Table 1, step 6, Fig 1C) and the body mass constraints associated with heat-dissipation time (Table 1, step 7). The simple *metabolic model* implicitly assumes that all animals dedicate their total time budget, *t*_*total*_ (*s*), exclusively towards locomotion and, therefore, travel at speeds that minimise their absolute metabolic cost of locomotion. This yields a power-law scaling of realised travel speed with body mass (Table 1, step 8 first column, Fig 1D). Both heat-dissipation models assume that the total time budget is not entirely dedicated towards locomotion as travelling animals allocate some time, *t*_*diss*_ (*s*), towards the dissipation of metabolic heat—an inevitable by-product of muscular contractions (Table 1, step 6). Rather than accelerating and decelerating from rest, we assume that animals allocate heat-dissipation time at small time-steps throughout the locomotion process, for example, between each stride (conceptualised in Fig 1A, lowest bar). An increase in heat-dissipation time, therefore, corresponds to a reduced stride frequency and a decrease in the realised travel speed that can be sustained. Our second model, the *constant heat-dissipation model*, is a saturating (nondecreasing) allometric scaling model (Fig 1D) that assumes, implicitly, that all animals possess the necessary physiological and/or behavioural adaptations to adequately facilitate the dissipation of metabolic heat via body mass–independent pathways (Table 1, steps 7 to 8 middle column). Our final model, the *allometric heat-dissipation model*, is a hump-shaped allometric scaling model (Fig 1D). It includes the additional assumption that the maximum heat-dissipation capacity of animals, and thus, the additional time that must be allocated towards heat dissipation, also scales with body mass (Table 1, step 7 right column). This implies that larger animals have greater thermal inertia—their body temperature changes more slowly than that of smaller animals when experiencing the same thermal gradient. Accordingly, larger animals require more time to dissipate the heat that is produced while moving a unit distance. A consequence of this body mass–dependent allocation of additional heat-dissipation time is that the largest animals must experience a net reduction in their travel speed in order to effectively regulate their body temperature during the extended locomotion bouts. This yields a hump-shaped scaling of realised travel speed with body mass (Table 1, step 8 right column, Fig 1D).

## Results

We compared the ability of 3 hypothesis-driven models (see Table 1) to predict the travel speeds of animals across 3 different modes of locomotion. Model comparison using LOOIC showed that the *allometric heat-dissipation model* (Table 1, step 8) best describes the systematic relationship between body mass and realised travel speed across flying, running, and swimming animals while the *metabolic model* and the *constant heat-dissipation model* scored substantially worse (Table 2).

**Table 2:** Comparison of three alternative allometric locomotion models that predict the realised travel speeds of animals as a function of their body mass and locomotion mode.

Model	Description	LOOIC	ΔLOOIC	SE ΔLOOIC
Metabolic	Power law	465.4	64.1	14.8
Constant heat-dissipation	Saturating	428.4	27.1	6.2
Allometric heat-dissipation	Hump-shaped	401.3	0.0	0.0

LOOIC values are presented together with the difference in LOOIC value relative to the most parsimonious model (ΔLOOIC = 0.0) and the estimated standard error of the difference (SE ΔLOOIC). All models fit the locomotion rate constant, *v*_0_, independently (i.e., no pooling) for flying, running, and swimming animals. The data underlying this Table can be found in https://zenodo.org/record/7554842

**Fig 2:**
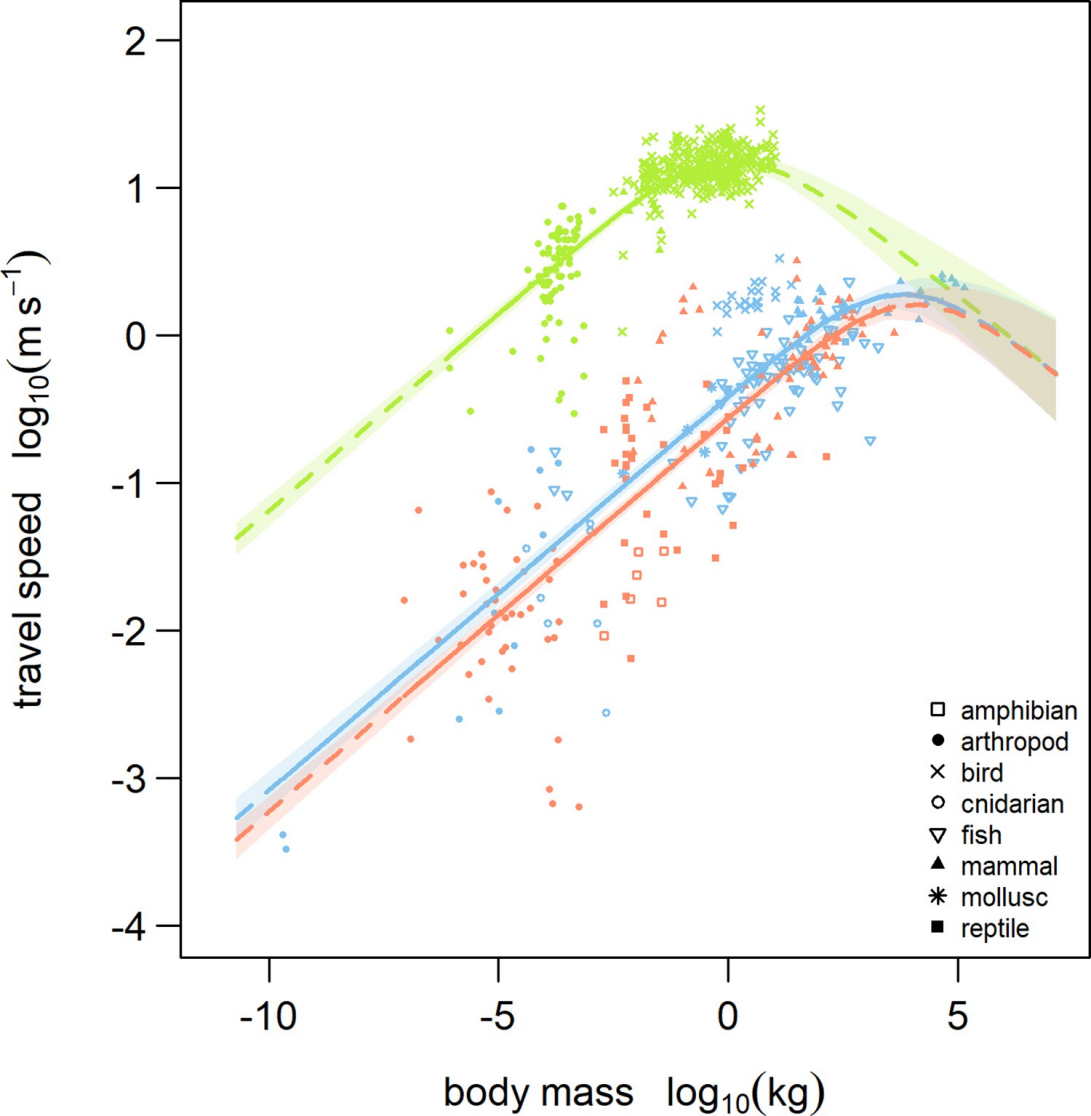
Realised travel speed as a function of body mass and locomotion mode as predicted by the *allometric heat-dissipation model*. Model-predicted mean values and 90% credible intervals are shown for flying (green), running (red), and swimming (blue) animals. The locomotion rate constant, *v*_0_, is fitted independently (i.e., no pooling) for each locomotion mode. Solid lines are predictions from the empirically observed range of body masses within each respective locomotion mode, and dashed lines are predictions extrapolated beyond that range. The data underlying this Figure can be found in https://zenodo.org/record/7554842.

The *allometric heat-dissipation model* predicts 3 hump-shaped relationships (in log-log space) that, by accounting for differences in the locomotion rate constant, *v*_0_, among locomotion modes, describe the realised travel speeds of all flying, running, and swimming species as a function of their body mass (Fig 2 and Table 3). We tested more complex formulations of our *constant heat-dissipation model* and *allometric heat-dissipation model* that consider whether the higher heat-dissipation capacity afforded to animals moving within the aquatic realm (water) as opposed to within the terrestrial realm (air) would result in higher realised travel speeds among the largest swimming animals. Although both models had comparable prediction accuracies to that of our best-performing model (S1 Table), they both yielded the unrealistic prediction of lower heat-dissipation capacity within the aquatic realm (parameters *k*_0_ and *k*_*λ*_ in S2 and S3 Tables, respectively), which corresponded to the prediction of higher realised travel speeds among the largest terrestrial animals (S1 and S2 Figs). We also tested a slightly more complex formulation of the *allometric heat-dissipation model* that accounts for variation in the allometric scaling exponent *c* across the 3 locomotion modes (S3 Fig). This more complex model also yielded comparable prediction accuracies to that of the best-performing model (S1 Table). We have, therefore, focused on the results of the more parsimonious *allometric heat-dissipation model*, which also revealed important differences in travel speed across locomotion modes. On average, flying animals can sustain potential travel speeds that are 100 times greater than those of running and swimming animals of equivalent body mass, while the potential travel speeds of swimming animals are only marginally faster than those of running animals (parameter *v*_0_ in Table 3). Flying animals’ higher potential travel speed, however, leads to the earlier saturation and subsequent decrease in their realised travel speed with increasing body mass (Fig 2).

**Table 3:** Posterior parameter estimates of the best-performing allometric locomotion model, the *allometric heat-dissipation model* (see Tables 2 and S1).

Parameter	Mean	SD	5%	95%
*v*_0 (*flying*)_	30.54	2.58	26.56	35.07
*v*_0 (*running*)_	0.28	0.02	0.25	0.31
*v*_0 (*swimming*)_	0.39	0.03	0.34	0.43
*k*_*λ*_	0.033	0.005	0.026	0.041
*c*	0.27	0.01	0.26	0.28
*d*	0.24	0.03	0.19	0.29

Table entries correspond to the mean, standard deviation (SD), and 90% credible intervals of the model parameters’ posterior distributions (see Table 1 for a description of the model parameters). The data underlying this Table can be found in https://zenodo.org/record/7554842.

The *allometric heat-dissipation model* incorporates 2 allometric scaling exponents that characterise the body mass constraints on (1) metabolic power input and locomotion efficiency (*c* in Tables 1 and 3) and (2) heat-dissipation time (*d* in Tables 1 and 3). The full model derivation (equations 2–6 and 20–27 in S1 Text) yields the expected ranges for these scaling exponents according to the underlying processes: The allometric scaling exponent, which describes the initial increase in potential travel speed with increasing body mass, and thus, larger animals’ greater capacity to supply metabolic power combined with their higher locomotion efficiency, fell within the range of these theoretical expectations (expected: 0.01 < *c* < 0.33; observed: *c* = 0.27, 90% CI 0.26 to 0.28). Similarly, the allometric scaling exponent that describes the body mass dependence of heat-dissipation time, and thus, larger animals’ greater thermal inertia, also fell within the range of values derived from theoretical and empirical estimates of maximal heat-dissipation capacity (expected: 0.01 < *d* < 0.37; observed: *d* = 0.24, 90% CI 0.19 to 0.29). Together, these results support the conclusion that these different allometric scaling processes jointly influence the realised travel speeds of animals.

## Discussion

We found a hump-shaped scaling relationship of travel speed with body mass across running, flying, and swimming animals, which we explain using fundamental biophysical constraints on the supply, utilisation, and dissipation of energy and heat, as incorporated within our *allometric heat-dissipation model*. This leads to 2 general insights about the fate of metabolic energy that goes towards the production of mechanical work and that which is dissipated as heat: First, despite possessing the metabolic potential to sustain higher travel speeds, the realised travel speeds of the largest animals are limited due to the risk of hyperthermia. Second, flying animals sustain a higher metabolic power input and higher travel speeds and, therefore, begin to limit their realised travel speeds at smaller body masses than running or swimming animals that travel more slowly. By jointly considering how allometric constraints shape metabolic demands as well as the dissipation of heat, we can provide generalised predictions of animal travel speeds across different locomotion modes and ecosystem types when only the body mass is known.

Currently established models of animal locomotion typically produce power-law scaling relationships between locomotion speed and body mass by considering the dominance of a particular set of biophysical constraints on one critical aspect of the locomotion process; for example, biomechanical safety factors and the risk of physical injury [26,47], dynamic similarity in locomotor mechanics and stride length [27,48], and the availability of metabolic power [35,49]. The scientific elegance of these biophysical models is that they relate a real-world phenomenon such as the speed of animal locomotion to the first principles of physics and morphology. Nevertheless, one of the limitations shared by most power-law models has been their limited ability to generalise predictions of animal locomotion speeds across a sufficiently wide range of body masses and across taxonomic groups that vary considerably in their body plan and mode of locomotion (e.g., [35,50–52]). Although such models describe how a particular biophysical constraint influences the utilisation of energy by the locomotory musculature, they do not take into account the considerable fraction of the total metabolic demand that is dissipated internally as heat. This leads to predictions such as those of the power-law model in our study (the *metabolic model*), which, by overestimating the travel speeds of the largest animals, fails to capture the empirical data’s hump-shaped trends in log-log space. To address this, existing power-law models report values of the allometric scaling exponent, which are inconsistent across taxonomic groups and different body mass ranges: One pattern that emerges across studies is the tendency for larger scaling exponents to be reported within groups of small-bodied animals such as arthropods [38,39,53], whereas large-bodied animals such as vertebrates tend to exhibit scaling relationships with smaller [22,37,49], mass-independent [54,55], or even negative scaling exponents [37]. We have developed a biophysical model that reconciles these idiosyncrasies by incorporating both the allometry of locomotion efficiency (i.e., larger animals have a lower metabolic cost of locomotion) and the allometry of heat-dissipation capacity (i.e., larger animals require more time to dissipate metabolic heat), which, together, explain the initial increase, saturation, and inevitable decrease in realised travel speed with increasing body mass across each locomotion mode. The hump-shaped scaling relationship predicted by the *allometric heat-dissipation model* captures these trends in travel speed across the full range of animal body masses in our empirical dataset (from 2.00 × 10^−10^ to 140,000 *kg*), thereby setting universal limits to the sustained speed of any dispersing animal that relies on cyclical muscle contractions to fuel the performance of mechanical work.

The overall hump-shaped trend in the allometric scaling of travel speed is well supported in our data by the travel speeds recorded among the world’s largest animals across various ecosystem types. For example, the median cruising speeds (± SD) of fin whales (2.40 ± 0.52 m/s, 74,000 *kg*) and blue whales (2.08 ± 0.46 m/s, 140,000 *kg*) reported by Gough and colleagues [55] agree much more closely with the predictions of our *allometric heat-dissipation model* (fin whale: 1.62 m/s, 90% PI 0.45 to 5.82; blue whale: 1.46 m/s, 90% PI 0.42 to 5.31) than with those of the *metabolic model* (fin whale: 3.75 m/s, 90% PI 1.23 to 15.72; blue whale: 5.15 m/s, 90% PI 1.42 to 17.90). Moreover, the assumption that heat dissipation is a constraint to sustained locomotion also corresponds to some well-documented forms of behavioural thermoregulation. For example, most migrating birds fly at high altitudes or at night in colder air, which, in turn, reduces evaporative water losses [56,57]. These behaviours are associated with a lower oxygen availability and an increased mechanical cost of flight at altitude [58,59] or the failure to settle at suitable stopover sites (night flights; [60]) and, therefore, only remain beneficial when heat dissipation is critical to sustained locomotion. There is also evidence across various taxonomic groups that the endogenous heat produced during locomotion can contribute towards thermoregulatory requirements, for example, by allowing small mammals and birds to offsets a portion of the thermoregulatory cost associated with activity in colder climates [61,62] or by facilitating a form of intermittent endothermy among some of the largest flying insects such as sphinx moths [63,64]. Together, these examples illustrate the importance of metabolic heat production and dissipation for animals engaging in sustained movement behaviours such as exploration, dispersal, and migration, which lies at the core of our new model of aerobic travel speed.

Interestingly, a similar hump-shaped relationship has also been shown to characterise the scaling of maximum speed with body mass by considering the combination of a finite time available for acceleration, which is limited due to the restricted availability of energy stored in the muscle cells to fuel anaerobic metabolism, together with body mass constraints on animals’ energy storage capacity [41]. Surprisingly, this suggests that maximum speed and travel speed, although both hump-shaped in relation to body mass, could nevertheless be constrained by very different physiological processes that take precedence during short anaerobic bouts and sustained aerobic activity, respectively. This discrepancy highlights the importance of ecological context for understanding the processes that limit the performance of animals in different behavioural states.

Consistent with previous models [38,65], we show that travel speed initially (i.e., at low body masses) scales with an allometric exponent close to 0.27. This allometric scaling exponent emerges from the product of the allometries of maximal aerobic metabolism (scaling with an exponent between 0.80 and 0.97; [66–69]) and maximum locomotion efficiency (scaling with an exponent of approximately −0.67; [30,31,33]). While our statistical approach does not allow us to disentangle the relative contribution of these 2 interacting processes, the expected value of the exponent (between 0.13 and 0.30) agrees well with the empirically determined value of 0.27, supporting our model assumptions. This allometric scaling relationship holds until it reaches a saturation phase that characterises the maximum travel speeds that can be realised within each locomotion mode. Surprisingly, we found that this saturation phase in realised travel speeds with increasing body mass occurred much sooner in flying animals, between 0.1 kg (e.g., common starling) and 1 kg (e.g., herring gull), than in running or swimming animals (both saturating between 1,000 and 10,000 kg, e.g., Elephant or Orca). Instead of being attributable to the thermophysical properties of their respective realms (aquatic versus terrestrial), this discrepancy may be explained by the fact that flying animals sustain higher rates of aerobic metabolism during locomotion than running and swimming animals [59,70] and utilise muscles that operate with lower mechanical efficiencies [71,72]. Consequently, they encounter the limits of their allometric heat-dissipation capacity at a smaller body mass.

Following a saturation with increasing body mass, the realised travel speeds of the largest animals ultimately decrease with an allometric exponent of −0.24 across all locomotion modes. This arises as a consequence of the allometric scaling of heat-dissipation time (scaling as 0.24, 90% CI 0.18 to 0.29) and corresponds well with the assumptions of our *allometric heat-dissipation model* (equations 20–27 in S1 Text), which indicate that the largest flying, running, and swimming animals must travel more slowly to avoid hyperthermia. This is well illustrated by the observation that many large flying birds sustain flight speeds that are lower than those that would maximise their migration range or aerobic efficiency [73]. Nevertheless, the discrepancy between the observed scaling exponent for heat-dissipation time and the upper bound of our theoretical expectations (scaling as 0.37) suggests that thermoregulation during locomotion is not exclusively explained by allometric heat-dissipation constraints; physiological (e.g., counter-current vascular exchange; [74]) and behavioural (e.g., nocturnal activity; [75]) adaptations, as well as factors that increase convective heat transfer also potentially contribute towards reducing the downward curvature of our *allometric heat-dissipation model* predictions. The latter include relative humidity, wind speed, as well as the increased movement of air or water resulting from movement of the animal (itself a function of travel speed). Although analytical models such as ours, which are based on a simplified concept of thermal conductance (via Newton’s law of cooling), do not adequately accommodate the variety of thermoregulatory pathways considered by more detailed heat exchange models (e.g., [76]), they provide broad, quantitative predictions that characterise the responses of species even when the details of their biology and ambient environment are unknown.

In addition to the general similarity in the hump-shaped scaling relationship across locomotion modes, our study also quantifies important differences between running, flying, and swimming animals. Overall, flying animals are able to sustain much greater speeds than running or swimming animals of equivalent body mass. This is driven by an almost 100-fold larger value of their locomotion rate constant, *v*_0_, which encompasses the mass-independent interaction between the rate of aerobic metabolism and locomotion efficiency. Our model does not assume that metabolic power input and locomotion efficiency vary independently of one another [71], but rather, that there are systematic differences among flying, running, and swimming animals in their maximal capacities to (i) supply their muscles with metabolic energy via aerobic pathways [59,70] and (ii) utilise this energy efficiently during locomotion [30,31,33]. Overall, our *allometric heat-dissipation model* not only predicts the hump-shaped relationship between realised travel speed and body mass but also provides an explanation for the differences in the shape of this scaling relationship between locomotion modes.

We have derived the *allometric heat-dissipation model* from physical first principles based on the core mechanistic components of (1) metabolic energy supply, (2) the metabolic cost of locomotion, and (3) heat-dissipation capacity. The fit of our model to empirical data yielded a general parameterisation that will allow for future predictions of animal movement capacities based on body mass and locomotion mode as the only species traits. The simplicity of the model structure and generality of its applicability come at the expense of excluding additional constraints that may affect the speed of locomotion without universally affecting any of the 3 core mechanistic model components: This includes, for example, morphology (e.g., limb length, hovering versus forward flapping flight), phylogenetic history, or thermoregulatory strategy. While the inclusion of these covariates could improve the prediction of travel speeds for specific groups of animals, it would come at the expense of generality in model predictions across all locomotion modes. For example, species’ limb length could be added to better predict the travel speed of running animals [32,77], whereas this component would be less meaningful when considering the effects of lift and drag on flying and swimming animals. We chose to exclude information on phylogenetic relatedness because the biophysical principles included in our model are deeply rooted in evolutionary history (e.g., total mitochondrial volume, protein temperature dependence) and should therefore apply to all taxonomic groups. Similarly, we chose to exclude thermoregulatory strategy as a categorical covariate due to the universal nature of animals’ locomotory demands (scaling with an exponent between 0.80 and 0.97; [66–69]), which, during periods of sustained aerobic activity, are responsible for more than 90% of metabolic heat production (see S1 Text). Their inclusion is further complicated by the problem of “confounding by cluster” [78] whereby body mass is not sufficiently represented across a wide enough range within each level of the categorical covariate to fit the relationship. From a philosophical perspective, the inclusion of phylogenetic or thermoregulatory covariates to improve the model fit would defeat the purpose of developing a universal model based on biophysical first principles. Therefore, we have currently limited our approach to biophysical processes that can be generalised across all modes of locomotion and across taxonomic groups.

There is scope for additional environmental or morphological factors that affect any of the 3 core mechanistic model components to be included as extensions of our model; their potential importance can be illustrated by analysing deviations from our model predictions: For example, Arctic wolves (*Canis lupus*) on Ellesemere Island [79]—the most northerly island within the Arctic Archipelago—travel 1.1 *m s*^−1^ faster than the upper bound of the 90% prediction interval (PI) of our *allometric heat-dissipation model*. We anticipate that they are able to sustain such high speeds over distances of 2 to 4 km while returning to their summer dens because the ambient temperatures that they experience rarely exceed 5.0°C, thus enabling a more rapid dissipation of the heat that their muscles produce. This illustrates an important effect of low ambient temperature on reducing the time required for heat dissipation, which, in turn, increases the thermoregulatory capacity (of large animals) to sustain high travel speeds. In its current form, our *allometric heat-dissipation model* includes the simplifying assumption that core body temperature increases with distance travelled without specifically considering the temperature of the body or that of the ambient environment. Incorporating temperature data into our model could therefore further elucidate additional physiological limits to animals’ capacities for sustained locomotion. We also found that morphological defence traits such as shell of the Galapagos giant tortoise (*Chelonoidis niger*)—the largest extant terrestrial ectotherm—coincides with sustained speeds that are considerably slower than the lower bound of our model’s 90% PI. We propose that the incredibly slow walking speeds of Galapagos giant tortoises arise because of their shell’s limited capacity to dissipate endogenously produced heat rather than because of its weight [80]. This suggests an interesting trade-off between local persistence through the defence against natural enemies and the capacity to disperse to distant but (potentially) predator-free environments. Moreover, the evolution of morphological adaptations that facilitate heat dissipation (e.g., counter-current systems [74], the avian bill [81]) may have important implications for the movement capacities of animals in ancient and contemporary landscapes. Together, these examples illustrate how additional characteristics of a species’ morphology and its thermophysical environment could be incorporated into our *allometric heat-dissipation model*. The model, thereby, retains its generality across a wide range of taxonomic groups and locomotion modes by including the quantitative responses of model components such as heat-dissipation capacity to these characteristics.

## Conclusions

Animal movement plays a critical role in shaping ecological dynamics across spatial scales [82–84]. Realistic models of landscape-scale biodiversity dynamics must incorporate large numbers of species whose movement rates can be predicted only on the basis of easily quantifiable traits such as body mass and locomotion mode. Therefore, allometric locomotion models such as our *allometric heat-dissipation model* could provide the modular “building blocks” of dynamic metacommunity or meta-food web models (e.g., [7–9]). This has a great potential to reveal the interplay between spatial processes and species interactions in driving biodiversity patterns across spatial scales [9,17,85,86]. In contrast to existing power-law models [22,35–39], our *allometric heat-dissipation model* accurately predicts that sustained travel speed follows a hump-shaped relationship with body mass. With respect to the spatial processes linking local communities to one another within metacommunities such as forest or island archipelagos, the identification of the novel link between metabolic heat-dissipation constraints and animal movement capacity implies that large animal species may be more susceptible to the effects landscape fragmentation than previously anticipated [18,87]. Specifically, the larger total metabolic energy expenditure associated with increasing animal body mass needs to be balanced by an increased ability to track spatial resource dynamics at the landscape scale. Our results suggest that, as animal body mass increases beyond the critical threshold defined by the saturation phase of our locomotion model, further increases in total metabolic demand will coincide with a decreasing movement capacity. Consequently, the high extinction risk observed among large animals, especially terrestrial herbivores and reptiles [88], may also be driven by their inability to balance their metabolic demands by efficiently locating resources within patchy landscapes [89,90]. Our *allometric heat-dissipation model* helps to reconcile animal movement theory with empirical biodiversity patterns and underpins the novel call to protect large animals from the potentially dire consequences of landscape fragmentation.

## Materials and methods

### The dataset

Empirical estimates of sustained travel speed for flying, running, and swimming animals were obtained by searching the Web of Science Core Collection for published studies using the following keywords: (optimal OR cruising OR travel OR routine OR dispersal OR sustained) AND (velocit* OR speed* OR motilit*) AND (run* OR walk* OR terrestrial OR flight* OR fly* OR swim*). The asterisks are wildcard endings that broadened the search. Our initial literature search, which included studies published prior to January 2022 (16,305 records), was refined by only including papers from the Web of Science categories that were potentially related to animal ecology (Marine Biology, Entomology, Environmental Sciences, Molecular and Cell Biology, etc.). This yielded a total of 2,826 potentially useful records. We supplemented our search for underrepresented taxa by searching Google Scholar with various taxonomic terms and by searching the bibliographies of relevant publications for additional data sources.

We included data from field and laboratory studies that reported mean or median speeds of individual animals or groups of animals maintaining sustained and directed movements within an unrestrained setting. This precluded the use of movement data obtained from treadmills, flight mills, swim tunnels, wind tunnels, as well as from animals who were stimulated to move by an observer. Many of these excluded papers reported animals’ critical speeds or their maximal aerobic speeds. We included data from studies that estimated voluntary travel speeds either directly (i.e., instantaneously) through visual observations, video recordings, radar, and animal-attached devices or indirectly from higher-frequency (sampling interval <30 minutes) telemetry data. For flying animals, we only considered flight speeds during powered (i.e., thrust generated by flapping) flight. When individual- or species-level body mass was not provided, we referred to secondary literature sources to assign the average adult body mass of the species (e.g., [91]). In cases where only body length was given, we used published allometric equations to estimate the wet body mass (e.g., [92]). For studies that reported individual-level data, we aggregated data to the species level by calculating the unweighted geometric mean of individual travel speeds and, where available, individual body masses. We extracted data directly from the text and tables of publications or by using the open-source image analysis software ImageJ 1.52 (National Institute of Health, USA) to digitise published figures. This resulted in a dataset that featured 699 estimates of mean or median travel speed taken from 170 studies across a pool of 532 species from various taxonomic groups (amphibians, arthropods, cnidarians, birds, fishes, mammals, molluscs, reptiles) that spanned 15 orders of magnitude in body mass (from 2.00 × 10^−10^ to 140,000 *kg*) and 5 orders of magnitude in travel speed (from 3.3 × 10^−4^ to 33.6 *m s*^−1^). The underlying data and the code needed to reproduce the analyses can be downloaded from Zenodo (https://zenodo.org/record/7554842) [93].

### Model specification

We used Bayesian parameter estimation to evaluate the relationship between body mass and travel speed. Bayesian models are comprised of 3 components: (i) a stochastic data model that links model predictions to the observed data; (ii) a deterministic process model that describes each of our mechanistic hypotheses; and (iii) a parameter model that includes prior assumptions about the parameter values.

#### (i) Data model

For the data model, we assumed a Gaussian likelihood for the log_10_-transformed travel speed *v*_*i*_ (in m/s):

$${log}_{10}(v_{i})∼Gaussian(f(M_{i},\theta_{i}),\sigma )$$ (1)

where the process model, *f*(·), predicts expected travel speed from the observed body mass, *M* (in *kg*) the vector of observation-level parameters, *θ*_*i*_ = {*c*, *d*, *v*_0_, *k*_0_, *k*_*λ*_}, and the standard deviation, *σ*, between model predictions and the observed data.

#### (ii) Process model

We considered 3 alternative process models of varying complexity, which corresponded to our 3 alternative hypotheses (Table 1, step 8, and Fig 1D) about the form of the allometric scaling relationship for realised travel speeds. Each process model was reformulated in log_10_-linear form. We included locomotion mode as a categorical covariate by estimating the locomotion rate constant (parameter *v*_0_) independently (i.e., no pooling) for flying, running, and swimming animals; the normalisation constants associated with heat-dissipation time (*k*_0_ or *k*_*λ*_) did not vary among locomotion modes (i.e., complete pooling).

#### (iii) Parameter model

We specified weakly informative prior distributions in the parameter model. The observation-level parameters for the allometric scaling exponents *c* and *d* were assumed to have independent half-normal priors; observation-level parameters for the normalisation constants *v*_0_, *k*_0_, and *k*_*λ*_ were assigned independent gamma distribution priors. We assumed a half-Cauchy prior distribution for the observation-level variances.

### Model selection and inference

Model selection and inference included the evaluation of the alternative allometric process model formulations. We fitted each model using a No-U-Turn Hamiltonian Monte Carlo Sampler (NUTS-HMC) in Stan via the rstan package [94] in R 4.0.2 [95] by employing three parallel NUTS-HMC chains with an adaptation phase of 1,500 iterations and a sampling phase of 3,000 iterations each. This yielded a sum of 9,000 samples of the posterior distribution for each model. A visual assessment of the trace and density plots confirmed that the NUTS-HMC chains had mixed adequately; Gelman–Rubin statistics ≤1.01 and high (>1,000) effective sample sizes verified convergence [96]. We compared the out-of-sample prediction accuracies of each model by calculating the LOOIC (leave-one-out information criterion) from the expected log predictive densities (ELPDs) using the log-likelihood values of the posterior samples (R package loo; [97]). We used a difference in LOOIC (ΔLOOIC), which was larger than at least 2 times the estimated standard error of the difference (SE ΔLOOIC) to distinguish among competing models [98]. Samples from the posterior distribution were used to characterise the distribution of parameter values and to estimate model uncertainty by reporting central and 90% credible intervals (CIs) for parameter estimates as well central and 90% PIs from model-based predictions of realised travel speed.

## Supporting information

S1 Fig Predictions from the constant heat-dissipation model for realised travel speed as a function of body mass with the heat-dissipation time constant *k*_0_ fitted independently (i.e., no pooling) for animals moving within the aquatic and terrestrial realms (see S2 Table). Model-predicted mean values and 90% credible intervals are shown for flying (green), running (red), and swimming (blue) animals. The locomotion rate constant, *v*_0_, is fitted independently (i.e., no pooling) for each locomotion mode. Solid lines are predictions from the empirically observed range of body masses within each respective locomotion mode and dashed lines are predictions extrapolated beyond that range. The data underlying this Figure can be found in https://zenodo.org/record/7554842.
(TIFF)

S2 Fig Predictions from the allometric heat-dissipation model for realised travel speed as a function of body mass with the heat-dissipation time constant *k*_*λ*_ fitted independently (i.e., no pooling) for animals moving within the aquatic and terrestrial realms (see S3 Table). Model-predicted mean values and 90% credible intervals are shown for flying (green), running (red), and swimming (blue) animals. The locomotion rate constant, *v*_0_, is fitted independently (i.e., no pooling) for each locomotion mode. Solid lines are predictions from the empirically observed range of body masses within each respective locomotion mode, and dashed lines are predictions extrapolated beyond that range. The data underlying this Figure can be found in https://zenodo.org/record/7554842.
(TIFF)

S3 Fig Predictions from the *allometric heat-dissipation model* for realised travel speed as a function of body mass with the allometric scaling exponent *c* fitted independently (i.e., no pooling) for each locomotion mode. Model-predicted mean values and 90% credible intervals are shown for flying (green), running (red), and swimming (blue) animals. The locomotion rate constant, *v*_0_, is fitted independently (i.e., no pooling) for each locomotion mode. Solid lines are predictions from the empirically observed range of body masses within each respective locomotion mode, and dashed lines are predictions extrapolated beyond that range. The data underlying this Figure can be found in https://zenodo.org/record/7554842.
(TIFF)

S1 Table Comparison of eight alternative allometric locomotion models that predict the realised travel speeds of animals as a function of their body mass and locomotion mode. The more complex versions of each of the allometric locomotion models featured in Table 1 also allow for variation in the allometric scaling exponent *c* among flying, running, and swimming animals or for variation in the heat-dissipation time constants *k*_0_ or *k*_*λ*_ among the aquatic and terrestrial realms. LOOIC values are presented together with the difference in LOOIC value relative to the most parsimonious model (ΔLOOIC = 0.0) and the estimated standard error of the difference (SE ΔLOOIC). LOOIC represents the expected log pointwise-predictive densities (ELPDs) converted to the deviance scale. The asterisks highlight the joint best-fitting models whose difference in LOOIC (ΔLOOIC) is within 2 standard errors of the difference (SE ΔELPD) and, therefore, comparable in terms of predictive performance. The data underlying this Table can be found in https://zenodo.org/record/7554842
(DOCX)

S2 Table Posterior parameter estimates of the joint best-performing allometric locomotion model, the *constant heat-dissipation model* with shared slope and variable maxima (see S1 Table). Table entries correspond to the mean, standard deviation (SD), and 90% credible intervals of the model parameters’ posterior distributions (see Table 1 for a description of the model parameters). The data underlying this Table can be found in https://zenodo.org/record/7554842
(DOCX)

S3 Table Posterior parameter estimates of the joint best-performing allometric locomotion model, the *allometric heat-dissipation model* with shared slope and variable maxima (see S1 Table). Table entries correspond to the mean, standard deviation (SD), and 90% credible intervals of the model parameters’ posterior distributions (see Table 1 for a description of the model parameters). The data underlying this Table can be found in https://zenodo.org/record/7554842.
(DOCX)

S1 Text Model derivation. (DOCX)

## Abbreviations

CI: credible interval
ELPD: expected log predictive density
LOOIC: leave-one-out information criterion
NUTS-HMC: No-U-Turn Hamiltonian Monte Carlo Sampler
PI: prediction interval
