# Supplementary figures and images for: The travel speeds of large animals are limited by their heat-dissipation capacities
Source: PLoS Biol. 2023 Apr 18;21(4):e3001820. doi: 10.1371/journal.pbio.3001820 (PMC10112811; doi:10.1371/journal.pbio.3001820)

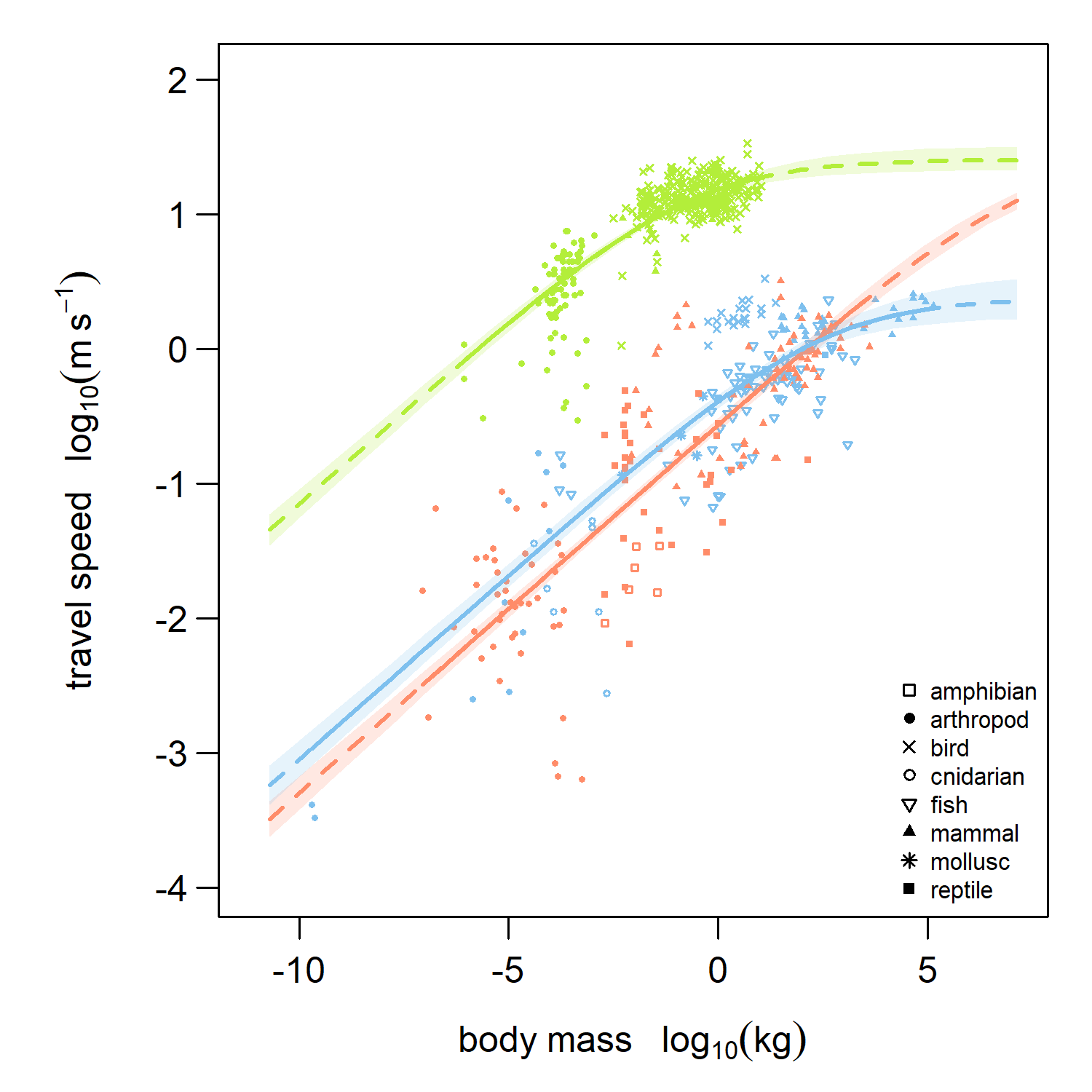

Supplement: S1 Fig — Model-predicted mean values and 90% credible intervals are shown for flying (green), running (red), and swimming (blue) animals. The locomotion rate constant, v0, is fitted independently (i.e., no pooling) for each locomotion mode. Solid lines are predictions from the empirically observed range of body masses within each respective locomotion mode and dashed lines are predictions extrapolated beyond that range. The data underlying this Figure can be found in https://zenodo.org/record/7554842. (TIFF) [file pbio.3001820.s001.tiff]

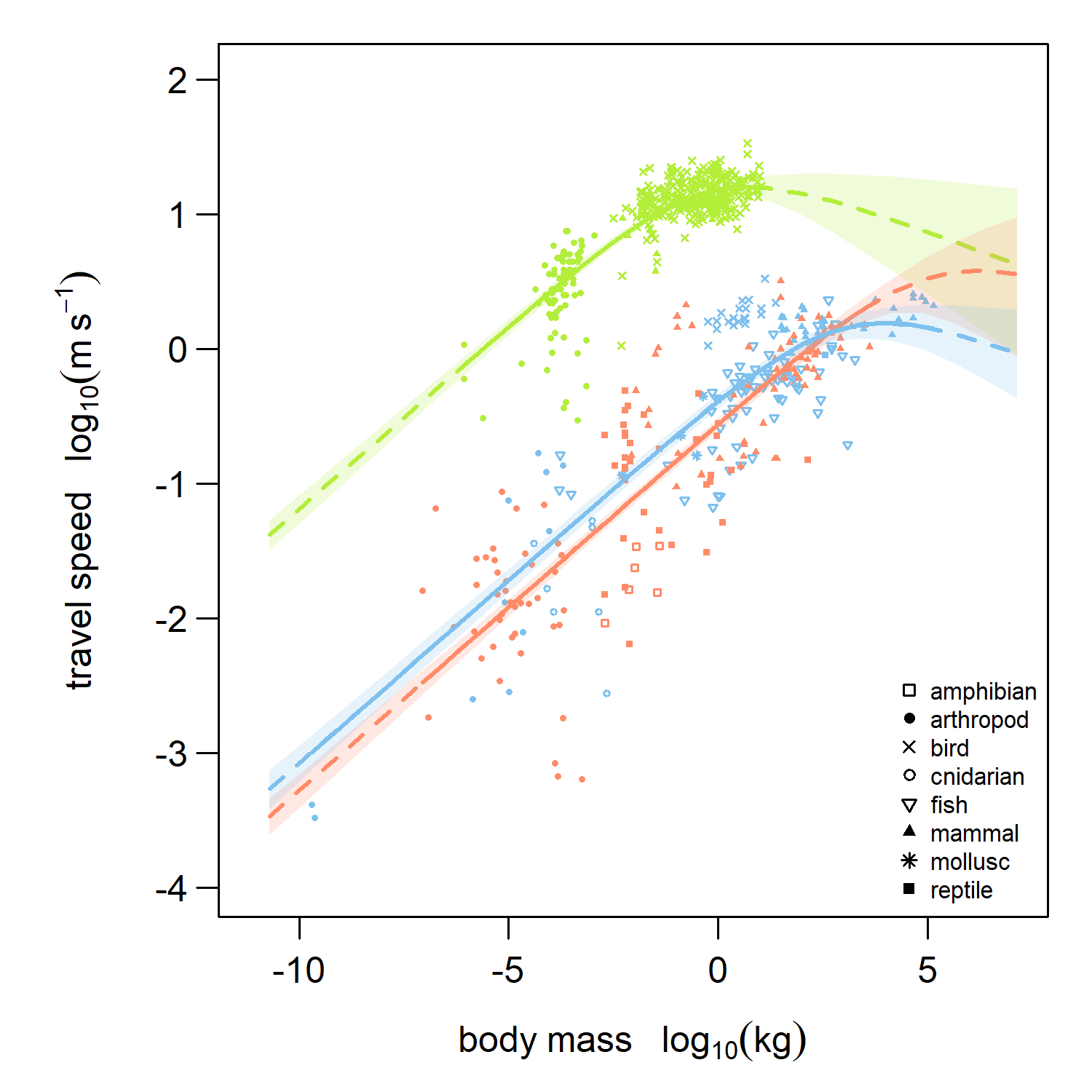

Supplement: S2 Fig — Model-predicted mean values and 90% credible intervals are shown for flying (green), running (red), and swimming (blue) animals. The locomotion rate constant, v0, is fitted independently (i.e., no pooling) for each locomotion mode. Solid lines are predictions from the empirically observed range of body masses within each respective locomotion mode, and dashed lines are predictions extrapolated beyond that range. The data underlying this Figure can be found in https://zenodo.org/record/7554842. (TIFF) [file pbio.3001820.s002.tiff]

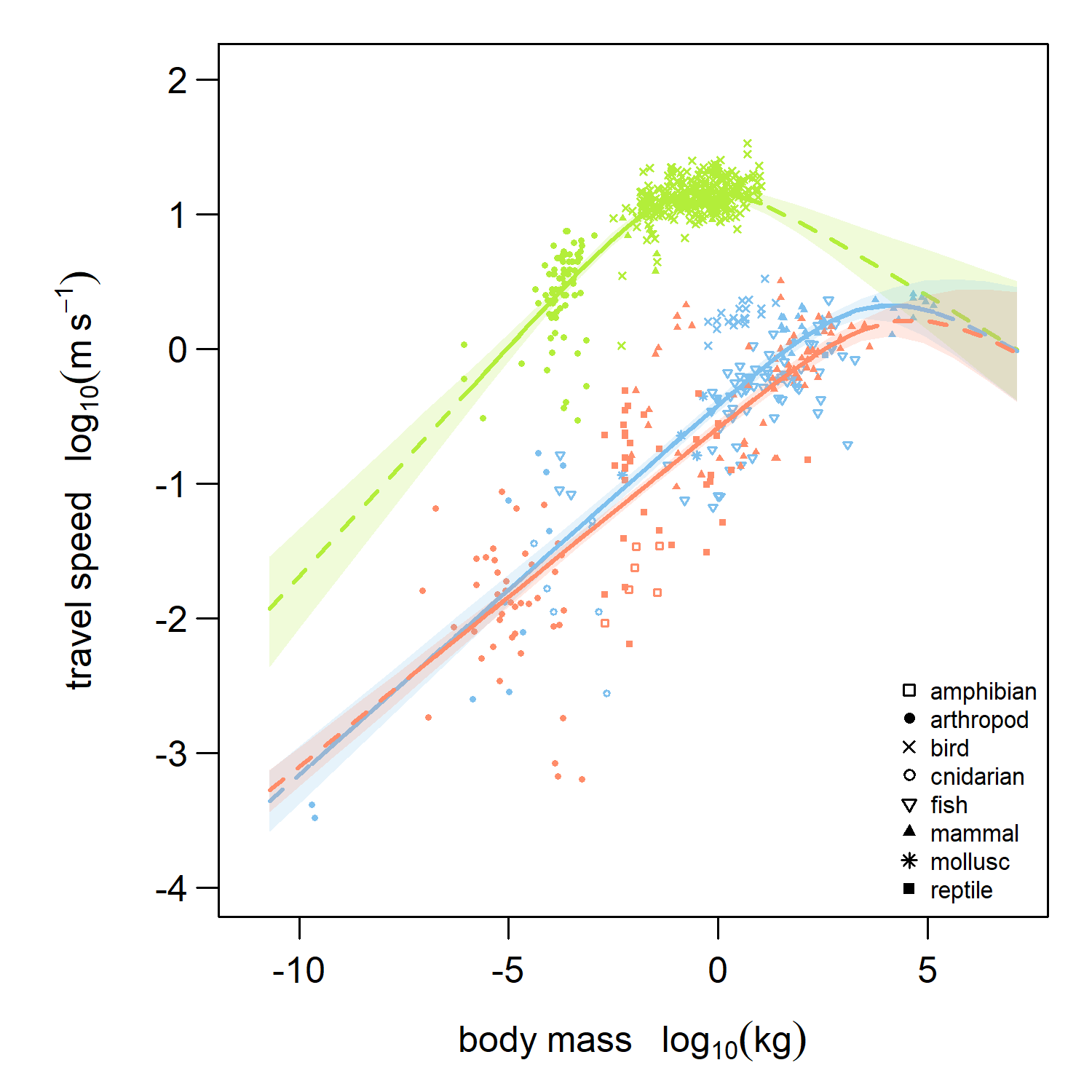

Supplement: S3 Fig — Model-predicted mean values and 90% credible intervals are shown for flying (green), running (red), and swimming (blue) animals. The locomotion rate constant, v0, is fitted independently (i.e., no pooling) for each locomotion mode. Solid lines are predictions from the empirically observed range of body masses within each respective locomotion mode, and dashed lines are predictions extrapolated beyond that range. The data underlying this Figure can be found in https://zenodo.org/record/7554842. (TIFF) [file pbio.3001820.s003.tiff]
