# Supplementary material for: The travel speeds of large animals are limited by their heat-dissipation capacities
Source: PLoS Biol. 2023 Apr 18;21(4):e3001820. doi: 10.1371/journal.pbio.3001820 (PMC10112811; doi:10.1371/journal.pbio.3001820)
Supplement: S1 Table — The more complex versions of each of the allometric locomotion models featured in Table 1 also allow for variation in the allometric scaling exponent c among flying, running, and swimming animals or for variation in the heat-dissipation time constants k0 or kλ among the aquatic and terrestrial realms. LOOIC values are presented together with the difference in LOOIC value relative to the most parsimonious model (ΔLOOIC = 0.0) and the estimated standard error of the difference (SE ΔLOOIC). LOOIC represents the expected log pointwise-predictive densities (ELPDs) converted to the deviance scale. The asterisks highlight the joint best-fitting models whose difference in LOOIC (ΔLOOIC) is within 2 standard errors of the difference (SE ΔELPD) and, therefore, comparable in terms of predictive performance. The data underlying this Table can be found in https://zenodo.org/record/7554842 (DOCX) [file pbio.3001820.s004.docx]

**S1 Table. Comparison of eight alternative allometric locomotion models that predict the realised travel speeds of animals as a function of their body mass and locomotion mode.** The more complex versions of each of the allometric locomotion models featured in Table 1 also allow for variation in the allometric scaling exponent $c$ among flying, running and swimming animals or for variation in the heat-dissipation time constants $k_{0}$ or $k_{\lambda}$ among the aquatic and terrestrial realms. LOOIC values are presented together with the difference in LOOIC value relative to the most parsimonious model (ΔLOOIC = 0.0) and the estimated standard error of the difference (SE ΔLOOIC). LOOIC represents the expected log pointwise-predictive densities (ELPD) converted to the deviance scale. The asterisks highlight the joint best-fitting models whose difference in LOOIC (ΔLOOIC) is within two standard errors of the difference (SE ΔELPD) and therefore comparable in terms of predictive performance. The data underlying this Table can be found in <https://zenodo.org/record/7554842>

| **Model** | **Description** | **LOOIC** | **ΔLOOIC** | **SE ΔLOOIC** |
| --- | --- | --- | --- | --- |
| Metabolic  (shared slope) | Power law:  only $v_{0}$ varies with locomotion mode | 465.4 | 65.0 | 14.5 |
| Metabolic  (variable slope) | Power law:  $v_{0}$ and $c$ vary with locomotion mode | 452.9 | 52.4 | 11.2 |
| Constant heat-dissipation  (shared slope) | Saturating:  only $v_{0}$ varies with locomotion mode | 428.4 | 28.0 | 8.4 |
| Constant heat-dissipation  (variable slope) | Saturating:  $v_{0}$ and $c$ vary with locomotion mode | 415.5 | 15.0 | 5.8 |
| Constant heat-dissipation  (shared slope and variable maxima) * | Saturating:  $v_{0}$ varies with locomotion mode and $k_{0}$ varies with ambient realm | 412.8 | 12.3 | 10.8 |
| Allometric heat-dissipation  (shared slope and variable maxima) * | Hump-shaped:  $v_{0}$ varies with locomotion mode and $k_{\lambda}$ varies with ambient realm | 404.1 | 3.6 | 8.7 |
| Allometric heat-dissipation  (shared slope) * | Hump-shaped:  only $v_{0}$ varies with locomotion mode | 401.3 | 0.9 | 5.6 |
| Allometric heat-dissipation  (variable slope) * | Hump-shaped:  $v_{0}$ and $c$ vary with locomotion mode | 400.5 | 0.0 | 0.0 |
