# Supplementary material for: The travel speeds of large animals are limited by their heat-dissipation capacities
Source: PLoS Biol. 2023 Apr 18;21(4):e3001820. doi: 10.1371/journal.pbio.3001820 (PMC10112811; doi:10.1371/journal.pbio.3001820)
Supplement: S3 Table — Table entries correspond to the mean, standard deviation (SD), and 90% credible intervals of the model parameters’ posterior distributions (see Table 1 for a description of the model parameters). The data underlying this Table can be found in https://zenodo.org/record/7554842. (DOCX) [file pbio.3001820.s006.docx]

**S3 Table. Posterior parameter estimates of the joint best-performing allometric locomotion model, the *allometric heat-dissipation model* with shared slope and variable maxima (see S1 Table).** Table entries correspond to the mean, standard deviation (SD), and 90% credible intervals of the model parameters’ posterior distributions (see Table 1 for a description of the model parameters). The data underlying this Table can be found in <https://zenodo.org/record/7554842>

| **Parameter** | **Mean** | **SD** | **5%** | **95%** |
| --- | --- | --- | --- | --- |
| $v_{0 (flying)}$ | 34.12 | 3.71 | 28.63 | 40.54 |
| $v_{0 (running)}$ | 0.28 | 0.02 | 0.25 | 0.31 |
| $v_{0 (swimming)}$ | 0.44 | 0.05 | 0.37 | 0.52 |
| $k_{\lambda(terrestrial)}$ | 0.037 | 0.005 | 0.029 | 0.044 |
| $k_{\lambda(aquatic)}$ | 0.179 | 0.092 | 0.057 | 0.354 |
| $c$ | 0.27 | 0.01 | 0.26 | 0.29 |
| $d$ | 0.12 | 0.05 | 0.03 | 0.21 |
