# Supplementary material for: The travel speeds of large animals are limited by their heat-dissipation capacities
Source: PLoS Biol. 2023 Apr 18;21(4):e3001820. doi: 10.1371/journal.pbio.3001820 (PMC10112811; doi:10.1371/journal.pbio.3001820)
Supplement: S1 Text — (DOCX) [file pbio.3001820.s007.docx]

# The travel speeds of large animals are limited by their heat-dissipation capacities

Alexander Dyer^1,2*^, Ulrich Brose^1,2^, Emilio Berti^1,2^, Benjamin Rosenbaum^1,2^, Myriam R. Hirt^1,2^

^1^ EcoNetLab, German Centre for Integrative Biodiversity Research Halle-Jena-Leipzig, Leipzig, Germany

^2^ Institute of Biodiversity, Friedrich‐Schiller‐University Jena, Jena, Germany

* Corresponding author

E-mail: dyer.alexander@protonmail.com

# S1 Text. Model derivation

Here we provide the detailed derivation of each of the three alternative allometric locomotion models. We consider throughout that the distance moved by an animal, $D$ ($m$) is equal to the product of (i) the rate of sustained metabolic power input (i.e. the active rate of aerobic metabolism), $P_{i}$($J s^{-1}$), (ii) the maximum locomotion efficiency, $E_{max}$($m J^{-1}$), and (iii) the time spent moving, $t_{move}$ ($s$).

$D= P_{i}{\cdot E}_{max}\cdot t_{move}$ (1)

## S1a. Metabolic model

### Metabolic power during sustained locomotion

Sustained locomotion is a cyclical process whereby metabolic energy reserves are utilised by the locomotory musculature to produce mechanical energy for the performance of work. Extended bouts of locomotion are sustained through the aerobic resynthesis (mitochondrial oxidative phosphorylation) of adenosine triphosphate (ATP) which depends, in turn, on the capacity of the vascular network to deliver an adequate supply of oxygen and metabolites to meet metabolic demands. The relationship between whole-organism metabolic rate, $Y$ ($Js^{-1}$), and body mass, $M$ ($kg$), is typically described by a power-law function of the form $Y={Y_{0}\cdot M}^{a}$ [1], where $a$ is the scaling exponent that describes how metabolic rate changes with body mass and $Y_{0}$ ($Js^{-1}{kg}^{-a}$) is a body mass-independent normalisation constant that characterises the relationship for a particular type of organism or taxonomic group.

The value of the allometric scaling exponent $a$ varies with the level of metabolic activity of the organism [2]: The lowest boundary constraint for $a$ is associated with the basal (i.e. minimum) metabolic rate required to maintain homeostasis which scales in the range between 0.67 and 0.75 [1,3-8]. The mechanistic basis for this relationship has been attributed to multiple processes, including surface area constraints on the flux of resources and metabolic by-products [5,9,10], physical constraints on the distribution of materials through branching vascular networks [11,12], and the optimisation of life-history traits [13]. Exercise-induced rates of aerobic metabolism scale more steeply with body mass than resting rates [2,4,5] (but see [14,15]). This coincides with the addition of locomotory demands which, during maximal aerobic activity, account for more than 90% of total oxygen demand [16,17]. Maximal rates of aerobic metabolism scale with body mass as $M^{0.80 to 0.97}$ [4,14,16,18] due to allometric constraints associated with maximum muscle force production (scaling as $M^{1.0}$, [19]), total muscle mass (scaling as $M^{1.0}$, [6,20]) or the total volume of muscle mitochondria (scaling as $M^{0.80 to 1.01}$, [16,21,22]). This sets the upper boundary for $a$ close to a value of 1.0. Accordingly, we assume that animals that are engaged in extended locomotion bouts (e.g. exploration, dispersal, migration) sustain rates of metabolic power input that scale with body mass according to the power function

$P_{i}=P_{0}\cdot M^{a}$ (2)

where $P_{0}$ ($Js^{-1}{kg}^{-a}$) is a normalisation constant and $a$ is an allometric scaling exponent ranging between 0.67 to 1.0.

### Metabolic cost of locomotion (locomotion efficiency)

The metabolic cost of locomotion, COL ($J kg^{-1}m^{-1}$), represents the energy required to move one unit of body mass one unit distance. It provides a gross measure of the efficiency with an animal’s locomotory musculature performs the work required for locomotion and includes the basal cost of metabolic maintenance. Measurements of respiration rates during locomotion show that COL is a non-linear decreasing function of speed in running animals [23,24] and a $u$-shaped function of speed in flying [25,26] (but see [27]) and swimming animals [28,29]. As a result, locomotion costs approach their minimum value, COL_min_ ($J kg^{-1}m^{-1}$), as running animals approach their maximum sustainable aerobic speed; similarly, flying and swimming animals approach COL_min_ while sustaining high aerobic speeds.

Despite comparative differences in the metabolic costs of flying, running and swimming, COL_min_ scales predictably with body mass (approximately as $M^{-0.33}$) across a wide range of animals that differ markedly in their mode of locomotion, body temperature, and skeletal architecture [23,30,31]. The higher locomotion efficiency attained by larger animals has been attributed to the effect of longer limbs on the rate of muscle force production (running, [32,33]) as well as the balance of fluid drag on locomotory appendages such as wings and fins (flying and swimming, [34,35]). As the whole-organism COL_min_ ($Jm^{-1}$) scales as $M^{0.67}$(=$M^{-0.33}\cdot M^{1})$, its reciprocal, maximum locomotion efficiency, $E_{max}$($mJ^{-1}$), scales according to the power function

$E_{max}=E_{0} {\cdot M}^{b}$ (3)

where $E_{0}$ ($mJ^{-1} {kg}^{-b}$) is the normalisation constant and $b$ is an allometric scaling exponent with an approximate value of -0.67.

### Realised travel speed

Substituting equations (2) and (3) into equation (1) yields an allometric relationship for travel distance:

$D={(P}_{0}\cdot E_{0})\cdot M^{a+b}\cdot t_{move}$ (4)

The metabolic model assumes that animals are only limited by energetic constraints during locomotion and, therefore, that $t_{move}=t_{total}$, where $t_{total}$ represents the total time budget available for travel. This assumption allows equation (4) to be simplified to yield an allometric relationship for travel distance

$D=v_{0}\cdot M^{c}\cdot t_{total}$ (5)

and realised speed

$v=\frac{D}{t_{total}}=v_{0}\cdot M^{c}$ (6)

which includes a combined normalisation constant, the locomotion rate constant, $v_{0}$ ($=P_{0}\cdot E_{0}$) ($m s^{-1} {kg}^{-c}$), that we assume varies by locomotion mode, and an allometric scaling exponent $c$ ($=a+b$) whose value ranges between 0.01 and 0.33.

## S1b. Constant heat-dissipation model

Heat is the by-product of muscular contractions and represents the fraction of the metabolic energy expended that is not converted into mechanical work. The muscles of swimming and running animals typically operate with peak mechanical efficiencies of between 15-42% [36,37] whereas the values reported among flying animals tend to be somewhat lower (insects: 3-17% [38-40], bats: 13% [41]), birds: 11-23% [26,42,43]). This implies that most of the metabolic energy expended during locomotion is dissipated as heat.

The heat produced during locomotion leads to an increase in core body temperature, $T$ ($K$), which influences the rates of physiological processes as well as the behaviours with which they are associated [44,45]. Although elevated body temperature initially leads to improvements in muscle performance and aerobic endurance [46-48], its unregulated increase inevitably impairs biomolecular stability and cellular functioning [49,50], contributing to the loss of locomotion capacity as animals approach their upper thermal limits [51-53]. In spite of this apparent thermal sensitivity, ectotherms and endotherms are capable of sustained locomotion across a wide range of ambient temperatures [54-57]: Ectotherms regulate their body temperatures through behavioural adaptations such as restricted activity times and the selection of favourable microhabitats [58,59], whereas endotherms achieve this primarily through physiological adaptations that facilitate counter-current heat exchange or evaporative- and non-evaporative heat loss [60,61]). We assume, more broadly, that the body temperature of any animal has to remain stable within its thermal limits during extended locomotion bouts; therefore, the rate of metabolic heat production associated with muscular work must be balanced by the rate at which the body dissipates heat to the ambient environment.

Newton’s law of cooling provides a simplified model of heat exchange that describes the rate of temperature change of a body as

$\frac{dT}{dt}=-k\cdot({T(t) - T}_{env})$ (7)

where $k$ is the Newtonian rate constant of temperature change ${(s}^{-1})$, $T(t)$ is the animal’s core body (i.e. muscle) temperature at time $t$ and $T_{env}$ is the ambient environmental temperature in Kelvin $(K)$. This implies that the instantaneous rate of change in an animal’s core body temperature is a constant fraction of the temperature difference between its core and the ambient environment. The rate constant $k$ can be determined either empirically, by measuring an animal’s heat-dissipation capacity under laboratory conditions, or calculated as the ratio of its thermal conductance, $C$ ($J s^{-1} K^{-1}$), to its body mass and the body’s specific heat capacity, $c_{p}$ ($J {kg}^{-1} K^{-1}$):

$k=\frac{C}{c_{p}\cdot M}$ (8)

By assuming the absence of significant radiant heat gain from the environment, an animal’s total heat flux, $H$ ($J s^{-1}$), can be approximated as a linear function of its thermal conductance and the thermal gradient that it experiences:

$H=C\cdot({T(t) - T}_{env})$ (9)

Our *constant heat-dissipation model* assumes that animals are not only limited by the metabolic demands of their locomotory musculature during locomotion; they also allocate additional time towards dissipating the metabolic heat that their muscles produce during locomotion. Consequently, $t_{total}= t_{move}+t_{diss}$, where $t_{diss}$ ($s$) represents the additional heat-dissipation time required for animals’ core body temperature to remain stable. Integrating equation (7) shows that, in the absence of additional metabolic heat production, the temperature difference between an animal’s core and its ambient environment ($T(t) {- T}_{env}$) decreases exponentially with time from a high initial value ($T_{0} {- T}_{env}$) towards thermal equilibrium with the ambient environment:

$T(t) {- T}_{env}= (T_{0}-T_{env})\cdot e^{-k\cdot t}$ (10)

Solving equation (10) for time $t$ yields the time required to dissipate the heat produced during locomotion:

$t_{diss}=\frac{1}{k}\cdot log\left( \frac{T_{0}-T_{env}}{T(t)-T_{env}} \right)$ (11)

where $T_{0}$ corresponds to the elevated core body temperature that has been reached by moving a unit distance. Here, we assume that core body temperature increases exponentially with distance moved:

$\frac{T_{0}-T_{Env}}{T(t)-T_{Env}} = e^{\alpha\cdot D}\Rightarrow log\left( \frac{T_{0}-T_{Env}}{T(t)-T_{Env}} \right) = \alpha\cdot D$ (12)

where $\alpha$ ($m^{-1}$) is a rate constant describing the relative temperature increase per unit distance. This assumption permits us to simplify the mathematical tractability of our model compared to more detailed heat-budget models (e.g. [62-65]) while still providing a general, yet realistic, mechanistic basis for including heat dissipation as a constraint to sustained locomotion speeds, even when the animal’s core body temperature and the temperature of its ambient environment are unknown. Combining equations (11) and (12) yields:

$t_{diss}=\frac{\alpha}{k}\cdot D$ (13)

$t_{diss}=k_{0}\cdot D$ (14)

where $k_{0}$ ($s$ $m^{-1}$) is the heat-dissipation time constant that we assume is body mass-independent if animals possess sufficient behavioural and physiological adaptations to facilitate thermoregulation during travel. This implies that specific heat capacity and thermal conductance are constants that do not vary significantly among species (equation 8). Our concept of heat-dissipation time does not require animals to expend the additional energy required to accelerate and decelerate from rest. Rather, time is allocated towards heat dissipation at small time-steps throughout the locomotion process (e.g. between each stride). Therefore, an increase in $t_{diss}$ corresponds to a net decrease in the realised travel speed. Notably, at small time-steps and short distances, the assumption that temperature increases exponentially with respect to the distance moved (equation 12) can be relaxed, as $e^{\alpha\cdot D}\simeq1+\alpha\cdot D$ (i.e. core body temperature increases linearly with distance moved [66,67]). Substituting $t_{move}=t_{total}-t_{diss}$ and equation (12) into equation (4), then solving for $D$, extends the simpler metabolic model and its assumptions (equations 1-6) to yield a non-linear relationship for distance moved

$D={(P}_{0}\cdot E_{0})\cdot M^{a+b}\cdot(t_{total}-t_{diss})$ (15)

$D=v_{0}\cdot M^{c}\cdot(t_{total}-k_{0}\cdot D)$ (16)

$D=\frac{v_{0}\cdot M^{c}\cdot t_{total}}{v_{0}\cdot M^{c}\cdot k_{0}+1}$ (17)

and realised travel speed:

$v=\frac{D}{t_{total}}=\frac{v_{0}\cdot M^{c}}{v_{0}\cdot M^{c}\cdot k_{0}+1}$ (18)

Rearranging equation (18) yields

$v=\frac{\frac{1}{k_{0}}\cdot M^{c}}{M^{c}+\frac{1}{v_{0}\cdot k_{0}}}$ (19)

which predicts a saturating increase in realised travel speed with increasing body mass for all values of the allometric scaling exponent $c$ between 0.01 and 0.33 with a normalisation constant, $v_{0}$, that varies across locomotion modes. The additional normalisation constant, $k_{0}$, is independent of body mass and locomotion mode and contains all of the variation (induced via behavioural and/or physiological adaptations) that is associated with the temperature gradient that exists between an animal’s body and its ambient environment during locomotion.

## S1c. Allometric heat-dissipation model

Heat-dissipation rates are unlikely to be body mass-independent due to the decrease in surface area to volume ratio that is associated with an increase in body mass. A broad collection of empirical data from both ecto- and endothermic taxa indicate that whole-organism thermal conductance, $C$ ($J s^{-1} K^{-1}$), increases as a power function of body mass (scaling between $M^{0.33}$ and $M^{0.62}$, summarised in [3]). This range of empirically-derived values for the allometric scaling exponent also correspond to more recent mechanistic model predictions of maximal heat-dissipation capacity in endotherms (scaling between$M^{0.47}$ and $M^{0.63}$) that consider the dissipation of metabolic heat from a core (e.g. muscle), across an insulating shell (e.g. pelage, adipose tissue layer or exoskeleton), to the ambient environment [68]. This implies that large animals will lose and gain heat more slowly than small animals when exposed to the same temperature gradient. The increase in thermal inertia which is associated with increasing body mass has several implications for the capacity of behavioural and physiological adaptations to offset the production of metabolic heat during sustained movement behaviours: larger species will require more heat-dissipation time and the largest species may have their capacity for sustained locomotion entirely restricted to colder ambient environments. Including an allometric scaling relationship for thermal conductance redefines our equation for $k$ (equation 8) to yield

$\lambda=\frac{C_{0}\cdot M^{d}}{c_{p}\cdot M}$ (20)

$\lambda={\lambda_{0}\cdot M}^{-d}$ (21)

where $\lambda$ ($s^{-1}$) is the rate constant of temperature change which scales negatively with body mass, $\lambda_{0}$ ($s^{-1} {kg}^{-d}$) is a normalisation constant which is independent of body mass and locomotion mode, and $d$ is the allometric scaling exponent. Combining equations (11) and (12) with equation (21) shows that $t_{diss}$ now scales positively with body mass:

$t_{diss}=\frac{\alpha}{\lambda_{0}}\cdot M^{d}\cdot D$ (22)

$t_{diss}=k_{\lambda}\cdot M^{d}\cdot D$ (23)

where $k_{\lambda}$ ($s m^{-1} {kg}^{-d}$) is the heat-dissipation time constant and $d$ is the allometric scaling exponent that falls within the range of 0.01 to 0.37. The upper boundary constraint for $d$ corresponds to the largest expected values of the allometric scaling exponent describing maximal heat-dissipation capacity across a wide range of taxonomic groups (scaling as $M^{0.63}$ [3,68]).

Our final model, the *allometric heat-dissipation model*, in addition to assuming that animals must allocate time towards the dissipation of metabolic heat during locomotion ($t_{move}=t_{total}-t_{diss}$), allows heat dissipation to scale negatively with animal body mass by accounting for the body mass-dependence of thermal conductance. Substituting $t_{move}=t_{total}-t_{diss}$ and equation (23) into equation (4), then solving for $D$, extends the simpler metabolic model and its assumptions (equations 1-6) to yield a non-linear relationship for distance moved

$D={(P}_{0}\cdot E_{0})\cdot M^{a+b}\cdot(t_{total}-t_{diss})$ (24)

$D=v_{0}\cdot M^{c}\cdot(t_{total}-k_{\lambda}\cdot M^{d}\cdot D)$ (25)

$v=\frac{D}{t_{total}}=\frac{v_{0}\cdot M^{c}}{v_{0}\cdot M^{c}\cdot k_{\lambda}\cdot M^{d}+1}$ (26)

which rearranges to

$v=\frac{\frac{1}{k_{\lambda}}\cdot M^{c}}{M^{c+d}+\frac{1}{v_{0}\cdot k_{\lambda}}}$ (27)

and predicts a hump-shaped relationship between realised travel speed and body mass for all realistic values of the allometric scaling exponents $c$ (0.01 to 0.33) and $d$ (0.01 to 0.37). Note that equation (27) initially saturates, attaining the highest realised travel speeds, before tending towards zero as body mass increases further beyond this point. This highlights a hard upper limit for the feasible body masses of animals capable of sustained locomotion in the absence of significant behavioural and/or physiological adaptations that further facilitate thermoregulation.

# References:

1. Kleiber M. Body size and metabolism. Hilgardia. 1932;6(11):315-53.

2. Glazier DS. A unifying explanation for diverse metabolic scaling in animals and plants. Biological Reviews. 2010;85(1):111-38.

3. Peters RH. The ecological implications of body size. 2nd ed. Cambridge, UK: Cambridge University Press; 1986.

4. White CR, Cassey P, Blackburn TM. Allometric exponents do not support a universal metabolic allometry. Ecology. 2007;88(2):315-23.

5. Glazier DS. Beyond the ‘3/4-power law’: variation in the intra- and interspecific scaling of metabolic rate in animals. Biological Reviews. 2005;80(04):611.

6. Calder WA. Size, function, and life history. Cambridge: Harvard University Press; 1984.

7. Rubner M. Über den Einfluss der Körpergröße auf Stoff- und Kraftwechsel. Zeitschrift für Biologie. 1883;19:535-62.

8. Brown JH, Gillooly JF, Allen AP, Savage VM, West GB. Toward a metabolic theory of ecology. Ecology. 2004;85(7):1771-89.

9. Sarrus PF, Rameaux JF. Application des sciences accessoires et principalement des mathématiques à la physiologie générale. Bull Acad R Méd. 1839;3:1094-100.

10. Dodds PS, Rothman DH, Weitz JS. Re-examination of the 3/4-law of Metabolism. Journal of Theoretical Biology. 2001;209(1):9-27.

11. West GB, Brown JH, Enquist BJ. A General Model for the Origin of Allometric Scaling Laws in Biology. Science. 1997;276(5309):122-6.

12. West GB, Brown JH, Enquist BJ. The Fourth Dimension of Life: Fractal Geometry and Allometric Scaling of Organisms. Science. 1999;284(5420):1677-9.

13. White CR, Alton LA, Bywater CL, Lombardi EJ, Marshall DJ. Metabolic scaling is the product of life-history optimization. Science. 2022;377(6608):834-9.

14. Gillooly JF, Gomez JP, Mavrodiev EV. A broad-scale comparison of aerobic activity levels in vertebrates: endotherms versus ectotherms. Proceedings of the Royal Society B: Biological Sciences. 2017;284(1849):20162328.

15. Gillooly JF, Allen AP. Changes in body temperature influence the scaling of and aerobic scope in mammals. Biology Letters. 2007;3(1):100-3.

16. Weibel ER, Hoppeler H. Exercise-induced maximal metabolic rate scales with muscle aerobic capacity. Journal of Experimental Biology. 2005;208(9):1635-44.

17. Hoppeler H, Weibel ER. Limits for oxygen and substrate transport in mammals. Journal of Experimental Biology. 1998;201(8):1051-64.

18. Killen SS, Glazier DS, Rezende EL, Clark TD, Atkinson D, Willener AST, et al. Ecological Influences and Morphological Correlates of Resting and Maximal Metabolic Rates across Teleost Fish Species. The American Naturalist. 2016;187(5):592-606.

19. Marden JH, Allen LR. Molecules, muscles, and machines: Universal performance characteristics of motors. Proceedings of the National Academy of Sciences. 2002;99(7):4161-6.

20. Alexander RM, Jayes AS, Maloiy GMO, Wathuta EM. Allometry of the leg muscles of mammals. Journal of Zoology. 1981;194(4):539-52.

21. Mathieu O, Krauer R, Hoppeler H, Gehr P, Lindstedt SL, Alexander RM, et al. Design of the mammalian respiratory system. VII. Scaling mitochondrial volume in skeletal muscle to body mass. Respiration Physiology. 1981;44(1):113-28.

22. Snelling EP, Seymour RS, Runciman S, Matthews PGD, White CR. Symmorphosis and the insect respiratory system: allometric variation. Journal of Experimental Biology. 2011;214(19):3225-37.

23. Taylor CR, Schmidt-Nielsen K, Raab JL. Scaling of energetic cost of running to body size in mammals. American Journal of Physiology. 1970;219(4):1104-7.

24. Full RJ, Zuccarello DA, Tullis A. Effect of variation in form on the cost of terrestrial locomotion. Journal of Experimental Biology. 1990;150(1):233-46.

25. Tucker VA. Bird Metabolism During Flight: Evaluation of a Theory. Journal of Experimental Biology. 1973;58(3):689-709.

26. Rayner JM. Estimating power curves of flying vertebrates. Journal of Experimental Biology. 1999;202(23):3449-61.

27. Ellington CP. Limitations on animal flight performance. Journal of Experimental Biology. 1991;160(1):71-91.

28. Beamish FWH. Swimming Capacity. In: Hoar WS, Randall DJ, editors. Fish Physiology. vol. 7. London, UK: Academic Press; 1978. p. 101-87.

29. O’Dor RK, Webber DM. Invertebrate athletes: trade-offs between transport efficiency and power density in cephalopod evolution. Journal of Experimental Biology. 1991;160(1):93-112.

30. Schmidt-Nielsen K. Locomotion: Energy Cost of Swimming, Flying, and Running. Science. 1972;177(4045):222-8.

31. Tucker VA. Energetic cost of locomotion in animals. Comparative Biochemistry and Physiology. 1970;34(4):841-6.

32. Pontzer H. A unified theory for the energy cost of legged locomotion. Biology Letters. 2016;12(2):20150935.

33. Kram R, Taylor CR. Energetics of running: a new perspective. Nature. 1990;346(6281):265-7.

34. Taylor GK, Nudds RL, Thomas ALR. Flying and swimming animals cruise at a Strouhal number tuned for high power efficiency. Nature. 2003;425(6959):707-11.

35. Floryan D, Buren TV, Smits AJ. Efficient cruising for swimming and flying animals is dictated by fluid drag. Proceedings of the National Academy of Sciences. 2018;115(32):8116-8.

36. Curtin NA, Bartlam-Brooks HLA, Hubel TY, Lowe JC, Gardner-Medwin AR, Bennitt E, et al. Remarkable muscles, remarkable locomotion in desert-dwelling wildebeest. Nature. 2018;563(7731):393-6.

37. Barclay CJ. Efficiency of Skeletal Muscle. In: Zoladz JA, editor. Muscle and Exercise Physiology. London, UK: Academic Press; 2019. p. 111-27.

38. Lehmann FO. The efficiency of aerodynamic force production in *Drosophila*. Comparative Biochemistry and Physiology Part A: Molecular & Integrative Physiology. 2001;131(1):77-88.

39. Ellington CP. The aerodynamics of hovering insect flight. VI. Lift and power requirements. Philosophical Transactions of the Royal Society of London B: Biological Sciences. 1984;305(1122):145-81.

40. Dickinson MH, Lighton JRB. Muscle Efficiency and Elastic Storage in the Flight Motor of *Drosophila*. Science. 1995;268(5207):87-90.

41. Norberg UM, Kunz TH, Steffensen JF, Winter Y, von Helversen O. The cost of hovering and forward flight in a nectar-feeding bat, *Glossophaga soricina*, estimated from aerodynamic theory. Journal of Experimental Biology. 1993;182(1):207-27.

42. Chai P, Chang AC, Dudley R. Flight thermogenesis and energy conservation in hovering hummingbirds. Journal of Experimental Biology. 1998;201(7):963-8.

43. Ward S, Möller U, Rayner JMV, Jackson DM, Bilo D, Nachtigall W, et al. Metabolic power, mechanical power and efficiency during wind tunnel flight by the European starling *Sturnus vulgaris*. Journal of Experimental Biology. 2001;204(19):3311-22.

44. Schulte PM, Healy TM, Fangue NA. Thermal Performance Curves, Phenotypic Plasticity, and the Time Scales of Temperature Exposure. Integrative and Comparative Biology. 2011;51(5):691-702.

45. James RS, Tallis J. The likely effects of thermal climate change on vertebrate skeletal muscle mechanics with possible consequences for animal movement and behaviour. Conservation Physiology. 2019;7(1):coz066.

46. Bennett AF. Thermal dependence of locomotor capacity. American Journal of Physiology-Regulatory, Integrative and Comparative Physiology. 1990;259(2):R253-8.

47. Full RJ, Tullis A. Capacity for sustained terrestrial locomotion in an insect: Energetics, thermal dependence, and kinematics. Journal of Comparative Physiology B. 1990;160(5).

48. Cheng AJ, Willis SJ, Zinner C, Chaillou T, Ivarsson N, Ørtenblad N, et al. Post-exercise recovery of contractile function and endurance in humans and mice is accelerated by heating and slowed by cooling skeletal muscle. The Journal of Physiology. 2017;595(24):7413-26.

49. Schulte PM. The effects of temperature on aerobic metabolism: towards a mechanistic understanding of the responses of ectotherms to a changing environment. Journal of Experimental Biology. 2015;218(12):1856-66.

50. Koch RE, Buchanan KL, Casagrande S, Crino O, Dowling DK, Hill GE, et al. Integrating Mitochondrial Aerobic Metabolism into Ecology and Evolution. Trends in Ecology & Evolution. 2021;36(4):321-32.

51. Payne NL, Smith JA, Meulen DE, Taylor MD, Watanabe YY, Takahashi A, et al. Temperature dependence of fish performance in the wild: links with species biogeography and physiological thermal tolerance. Functional Ecology. 2016;30(6):903-12.

52. Rezende EL, Bozinovic F. Thermal performance across levels of biological organization. Philosophical Transactions of the Royal Society B: Biological Sciences. 2019;374(1778):20180549.

53. Chung DJ, Schulte PM. Mitochondria and the thermal limits of ectotherms. Journal of Experimental Biology. 2020;223(20):jeb227801.

54. Tucker MA, Böhning-Gaese K, Fagan WF, Fryxell JM, Moorter BV, Alberts SC, et al. Moving in the Anthropocene: Global reductions in terrestrial mammalian movements. Science. 2018;359(6374):466-9.

55. Sunday JM, Bates AE, Dulvy NK. Global analysis of thermal tolerance and latitude in ectotherms. Proceedings of the Royal Society B: Biological Sciences. 2010;278(1713):1823-30.

56. Hirt MR, Barnes AD, Gentile A, Pollock LJ, Rosenbaum B, Thuiller W, et al. Environmental and anthropogenic constraints on animal space use drive extinction risk worldwide. Ecology Letters. 2021;24(12):2576-85.

57. Barbarossa V, Bosmans J, Wanders N, King H, Bierkens MFP, Huijbregts MAJ, et al. Threats of global warming to the world’s freshwater fishes. Nature Communications. 2021;12(1):1701.

58. Kearney M, Shine R, Porter WP. The potential for behavioral thermoregulation to buffer ‘cold-blooded’ animals against climate warming. Proceedings of the National Academy of Sciences. 2009;106(10):3835-40.

59. Pincebourde S, Woods HA. There is plenty of room at the bottom: microclimates drive insect vulnerability to climate change. Current Opinion in Insect Science. 2020;41:63-70.

60. Schmidt-Nielsen K. Countercurrent systems in animals. Scientific American. 1981;244(5):118-29.

61. Tattersall GJ, Arnaout B, Symonds MRE. The evolution of the avian bill as a thermoregulatory organ. Biological Reviews. 2016;92(3):1630-56.

62. Rubalcaba JG, Gouveia SF, Villalobos F, Cruz-Neto AP, Castro MG, Amado TF, et al. Physical constraints on thermoregulation and flight drive morphological evolution in bats. Proceedings of the National Academy of Sciences. 2022;119(15):e2103745119.

63. Stevenson RD. The Relative Importance of Behavioral and Physiological Adjustments Controlling Body Temperature in Terrestrial Ectotherms. The American Naturalist. 1985;126(3):362-86.

64. Speakman JR, Hays GC, Webb PI. Is Hyperthermia a Constraint on the Diurnal Activity of Bats? Journal of Theoretical Biology. 1994;171(3):325-39.

65. Powers DR, Tobalske BW, Wilson JK, Woods HA, Corder KR. Heat dissipation during hovering and forward flight in hummingbirds. Royal Society Open Science. 2015;2(12):150598.

66. Taylor CR, Lyman CP. Heat storage in running antelopes: independence of brain and body temperatures. American Journal of Physiology. 1972;222(1):114-7.

67. Hodgson DR, McCutcheon LJ, Byrd SK, Brown WS, Bayly WM, Brengelmann GL, et al. Dissipation of metabolic heat in the horse during exercise. Journal of Applied Physiology. 1993;74(3):1161-70.

68. Speakman JR, Król E. Maximal heat dissipation capacity and hyperthermia risk: neglected key factors in the ecology of endotherms. Journal of Animal Ecology. 2010;79:726-46.
