# Supplementary material for: The travel speeds of large animals are limited by their heat-dissipation capacities
Source: PLoS Biol. 2023 Apr 18;21(4):e3001820. doi: 10.1371/journal.pbio.3001820 (PMC10112811; doi:10.1371/journal.pbio.3001820)
Supplement: Attachment — Submitted filename: PBIOLOGY-D-22-01874_R1 - Review.pdf [file pbio.3001820.s008.pdf]

Comments to Author

MS ID: PBIOLOGY-D-22-01874R1

Title: Heat dissipation drives the hump-shaped scaling of animal dispersal speed with body mass

Major comments

This study developed a generalised biophysical model that integrates physiology in terms of metabolic heat dissipation with locomotor capacity to explain average speed across body size from a wide range of taxa. This is an interesting paper and I really enjoyed reading the logic and reasoning behind the model. The main conceptual critique is on the term "dispersal" used throughout the paper. While dispersal is ecologically important to understand how animals respond to a rapidly changing world, the general allometric model is looking at average movement (Table 1 step 1) and energy expenditure of locomotion (Table 1 step 3 and 4). I would recommend not limiting study to discussing dispersal because dispersal is a behavioural process or state of a one-way, long-distance movement to a new environment that is determined by intrinsic and extrinsic factors (Bowler & Benton 2005; Stevens *et al.* 2014; Wu & Seebacher 2022), which locomotor capacity (maximum speed, musculoskeletal capacity) and energy expenditure (metabolic rate, aerobic capacity) are one of the limiting factors for the rate of dispersal. In fact, the description of the data collection section indicates average speed from "*maintaining sustained and directed movements within an unrestrained setting*" was extracted which could indicate general activity or movement that can encompasses foraging, exploration, seeking shelter or mate, dispersal, or migration. There is no indication that the speeds extracted were specific to dispersal. Therefore, the study itself looks at how heat dissipation affects movement or locomotor capacity and not solely on dispersal. I would reframe the study to focus on locomotor capacity, with part of the argument that dispersal is an important component of locomotion. Therefore, the introduction, methods, results, and discussion should reflect this.

Bowler, D. E., & Benton, T. G. (2005). Causes and consequences of animal dispersal strategies: relating individual behaviour to spatial dynamics. *Biological Reviews*, 80, 205-225.

Stevens, V. M., Whitmee, S., Le Galliard, J. F., Clobert, J., Böhning-Gaese, K., Bonte, D., ... & Baguette, M. (2014). A comparative analysis of dispersal syndromes in terrestrial and semi-terrestrial animals. *Ecology Letters*, 17, 1039-1052.

Wu, N. C., & Seebacher, F. (2022). Physiology can predict animal activity, exploration, and dispersal. *Communications Biology*, 5, 1-11.

Results

L244-252: I disagree with this paragraph that the model did not include the covariables because they did not correspond to systematic deviations. This was a visual assessment and while there is large

variation between each covariates examined (in the supplementary figures), a sensitivity analysis or model comparison is highly recommended to statistically support the influence of these variables on the model output. Comparing these models therefore can provide confident when reasoning whether the covariables are necessary in the study.

Methods

To ensure standardised practice and transparency in reporting methods and results for comparative studies (Parker *et al.* 2018), I have included a "Transparency, openness, and reproducibility" checklist at the end of the document from the Tools for Transparency in Ecology and Evolution (TTEE; https://osf.io/g65cb/) website. This is to warrant appropriate methodological details reported in the study.

Parker, T. H., Griffith, S. C., Bronstein, J. L., Fidler, F., Foster, S., Fraser, H., Forstmeier, W., Gurevitch, J., Koricheva, J. and Seppelt, R. (2018). Empowering peer reviewers with a checklist to improve transparency. *Nature Ecology & Evolution* **2**, 929-936.

The methods section was straight forward and easy to follow. There were some important information missing from the data synthesis and analysis stage of the main document that requires further clarification:

- What was the total records from the Web of Science and Google Scholar search?
- How many studies were retained and excluded?
- What was the inclusion and exclusion criteria for keeping studies in the data.
- Provide the proper references in the raw data instead of numbers.
- Provide the *R* code for the analysis,
- Provide references for the *R* packages used and the *R* software in the main document.

Some additional caveats need to be acknowledged with the heat dissipation models, the model assumes the animal is constantly moving for the heat-dissipation model to function and ignores how many times the animal would need to rest. Under a hypothetical scenario it is fine but needs to be stated.

Tables

Table 1. Some missing descriptions of symbols such as P_0 and E_0, which I assume are the intercepts of the power function?

Figures

Fig 1: For part C - time budget, is the fraction of heat-dissipation relative to body size or total?

S1_Text

LS34-36: A recent study suggest metabolic-size scaling is due to evolutionary optimisation for life history (White et al. 2022), while another theory suggests the scaling relationship is due to the metabolic boundaries of cells (Glazier 2005, 2010). It would be worth considering when describing mechanistic basis for metabolic-size scaling relationships.

Glazier, D. S. (2005). Beyond the '3/4-power law': variation in the intra-and interspecific scaling of metabolic rate in animals. *Biological Reviews*, 80, 611-662.

Glazier, D. S. (2010). A unifying explanation for diverse metabolic scaling in animals and plants. *Biological Reviews*, 85, 111-138.

White, C. R., Alton, L. A., Bywater, C. L., Lombardi, E. J., & Marshall, D. J. (2022). Metabolic scaling is the product of life-history optimization. *Science*, 377, 834-839.

Eq 7: Is T in Celsius or Kevin?

Minor comments

L637: Journal missing in this paper.

Transparency openness, and reproducibility checklist

Category	Description	Checklist
**Introduction**		
Objectives/ research question(s)	Explicit statement of objectives, research questions and scope of study	Yes
**Methods**		
Scope of study	Clearly identify scope of the study (dates, locations, taxa, type of data)	Yes
Information sources	Describe all information sources (e.g., databases with dates of coverage, contact with study authors to identify additional studies) in the search and date(s) searched	Yes
Search criteria	Complete and explicit database search terms including all logical connectors and operators sufficient to exactly duplicate search. Also, indicate all other sources of data (e.g. literature cited sections of published reviews, personal unpublished data, collaborative group project data available from cited sources or online).	Incomplete
Study inclusion/ exclusion criteria	Document the specific criteria and methods for inclusion of studies at each stage of the selection process	No
Duplication of search and selection	State how searches were conducted and coded; did two independent observers conduct searches and code results (desirable if possible)?	No
Effect size measures	State and justify the measures of effect size used (e.g., Z-transform of correlation coefficient, standardized mean difference). If formulas for effect size and its sampling variance are not published elsewhere, provide sufficient information to calculate them and evaluate how they are used in analyses; describe their statistical properties and how the effect sizes were calculated or derived.	NA
Data extraction process	Describe how data were obtained from published or unpublished sources (e.g. figures digitized using cited software, correspondence with authors), and whether two or more independent observers duplicated data extraction	No
Data collection and processing	List and explain what data were collected even if they were not included in the final analysis (e.g. taxa, latitude/longitude, biomass) and any assumptions, simplifications or transformations made	Yes

Assessment of biases	Describe whether potential biases were assessed and if so, how this was done (e.g. publication bias, missing data or selective reporting within studies). Assessment could be formal quantitative analysis or narrative.	No
Weighting	Describe methods of weighting studies (e.g. inverse variance weighting) or statistical justification for not weighting	NA
Analysis	Delineate general approach to synthesis used (fixed-effect model, random-effects model or hierarchical/multilevel models), specific details of analyses (e.g., model used in meta-regressions with complete description of covariates, including methods used for calculating random effect variance), and assessment of assumptions of the analyses. Describe model selection if appropriate, and also types of analysis for publication bias and missing data, if applicable. Identify software used for all analyses.	Yes
Code	Describe full model specification and provide code or fully specify choices if software package with GUI is used (with citation)	Yes
Non-independence among study outcomes	Discuss possible sources of non-independence within and among studies and how they were handled (e.g., phylogenetic, time series, multiple analyses of the same database, same control group for multiple comparisons).	No
Other aspects of analyses	Describe how missing data were handled, full description of imputation methods if used, sensitivity analysis if used, etc.	NA
**Results**		
Results of study selection process	Provide numbers of studies screened and selected/excluded at each stage (with brief explanation, e.g., ‘wrong field’, ‘non-experimental’) and number of studies/cases included in final analysis, using Preferred Reporting Items for Systematic Reviews and Meta-Analyses (PRISMA)-like flowchart (www.prisma-statement.org)	No
Quantitative synthesis results	Summarize and synthesize results across studies, including central tendencies, regression coefficients and heterogeneity as appropriate, specifying confidence/credible intervals (e.g. 95%, 99%) around means and how they were calculated, number of studies included for each meta-analysis or component (e.g. subgroups), evaluation of heterogeneity with statistics reported (e.g. Q, I^2), results of sensitivity or other analyses (e.g., excluding a study with a large effect size) or other analyses (e.g., analysis for detecting publication bias and/or correcting for publication bias.	NA
Qualitative synthesis results	Summarize and synthesize results across studies; for systematic reviews without meta-analyses, present full categorization and evaluation of the literature and identify areas in which information (original research or published outcomes) is missing	Yes
**Discussion**		
Main findings	Evaluate and interpret the strength of evidence for the outcomes tested, including heterogeneity and contribution of covariates	NA
Possible biases	Evaluate and interpret potential sources and consequences of bias (e.g. publication bias, research bias in choice of systems studied)	NA

NA represents not applicable.
