# Supplementary material for: The travel speeds of large animals are limited by their heat-dissipation capacities
Source: PLoS Biol. 2023 Apr 18;21(4):e3001820. doi: 10.1371/journal.pbio.3001820 (PMC10112811; doi:10.1371/journal.pbio.3001820)
Supplement: Attachment — Submitted filename: DyerA_2023_ReponseToReviewers_Rev1.docx [file pbio.3001820.s009.docx]

**Editor:**

Thank you for your patience while your manuscript "Heat dissipation drives the hump-shaped scaling of animal dispersal speed with body mass" was peer-reviewed at PLOS Biology. It has now been evaluated by the PLOS Biology editors, an Academic Editor with relevant expertise, and by three independent reviewers.

In light of the reviews, which you will find at the end of this email, we would like to invite you to revise the work to thoroughly address the reviewers' reports.

As you will see below, the reviewers are broadly positive, but each has a number of requests that should be addressed before further consideration. For example, reviewer #1 asks that you re-frame the study away from “dispersal” and towards movement more generally (when asked to cross-comment, one other reviewer agreed strongly with this point), requests a sensitivity or model comparison, asks that you improve transparency of reporting (s/he provides a checklist), and wants some methodological clarifications and some caveats. Reviewer #2 asks you to explore the effects of convection, questions the interpretation of the data shown in Fig 2, and suggests that you also explore other variables, namely thermoregulatory mode (endothermy vs. ectothermy) and macrohabitat (aquatic vs. terrestrial).

**Authors:** We thank the editor and three referees for having taken the time to handle and review our manuscript and for facilitating a constructive review process. The comments by the reviewers were very insightful and have allowed us to improve our analyses as well as the structure of our manuscript: Following the suggestions we have made the following major revisions:

1. We have followed the suggestions by reviewer 1 and a second reviewer and reframed our study to focus more broadly on travel speed as an important component of animal movement capacity during extended (i.e. aerobic) bouts of various movement behaviours (e.g. exploration, dispersal, and migration).
2. We have addressed reviewer 1 and 2’s request to explore additional covariates by a model comparison with additional saturating and hump-shaped models that account for differences in thermal conductance experienced by animals moving in aquatic versus terrestrial realms. We have updated our methods and results to describe the process models that include this additional covariate, which also supports our interpretation of the results shown in Fig. 2.
3. We have also updated our methods to justify why we refrain from including thermoregulatory mode as a covariate within our allometric models and why it would detract from our research question whose focus is the development of a general allometric model, rather than a models that describe the allometries within particular groups (e.g. flying ectotherms, running endotherms, etc) whose body mass range is confounded by/clustered with thermoregulatory mode.
4. We have improved the transparency of reporting requested by reviewer 1 by completing the provided checklist, clarifying our data synthesis methodology, and uploading a reference list file and the code required to reproduce our results.

------------------------------------

**REVIEWERS' COMMENTS:**

**Reviewer #1:**

[IMPORTANT: See fully formatted review provided in the PDF]

Major comments:

This study developed a generalised biophysical model that integrates physiology in terms of

metabolic heat dissipation with locomotor capacity to explain average speed across body size from a

wide range of taxa. This is an interesting paper and I really enjoyed reading the logic and reasoning

behind the model. The main conceptual critique is on the term “dispersal” used throughout the paper.

While dispersal is ecologically important to understand how animals respond to a rapidly changing

world, the general allometric model is looking at average movement (Table 1 step 1) and energy

expenditure of locomotion (Table 1 step 3 and 4). I would recommend not limiting study to discussing

dispersal because dispersal is a behavioural process or state of a one-way, long-distance movement

to a new environment that is determined by intrinsic and extrinsic factors (Bowler & Benton 2005;

Stevens et al. 2014; Wu & Seebacher 2022), which locomotor capacity (maximum speed,

musculoskeletal capacity) and energy expenditure (metabolic rate, aerobic capacity) are one of the

limiting factors for the rate of dispersal. In fact, the description of the data collection section indicates

average speed from “maintaining sustained and directed movements within an unrestrained setting”

was extracted which could indicate general activity or movement that can encompasses foraging,

exploration, seeking shelter or mate, dispersal, or migration. There is no indication that the speeds

extracted were specific to dispersal. Therefore, the study itself looks at how heat dissipation affects

movement or locomotor capacity and not solely on dispersal. I would reframe the study to focus on

locomotor capacity, with part of the argument that dispersal is an important component of locomotion.

Therefore, the introduction, methods, results, and discussion should reflect this.

Bowler, D. E., & Benton, T. G. (2005). Causes and consequences of animal dispersal strategies: relating individual behaviour to spatial dynamics. Biological Reviews, 80, 205-225.

Stevens, V. M., Whitmee, S., Le Galliard, J. F., Clobert, J., Böhning‐Gaese, K., Bonte, D., ... & Baguette, M. (2014). A comparative analysis of dispersal syndromes in terrestrial and semi‐terrestrial animals. Ecology Letters, 17, 1039-1052.

Wu, N. C., & Seebacher, F. (2022). Physiology can predict animal activity, exploration, and dispersal. Communications Biology, 5, 1-11.

**Authors:** Thank you for this constructive assessment of our manuscript. It is promising to read that the reasoning behind our model assumptions came through clearly. We agree that our model and the data that we extracted from published studies apply more broadly to various forms of sustained (i.e. aerobic) movement behaviour, such as exploration, dispersal and migration. We have incorporated the suggestions by reframing our study to focus on ‘travel speed’ as a component of locomotor capacity, and locomotion capacity as an important constraint to movement behaviours such as exploration, dispersal, and migration. This revision has improved the description of our approach, which has broadened the scope of our manuscript.

L244-252: I disagree with this paragraph that the model did not include the covariables because they

did not correspond to systematic deviations. This was a visual assessment and while there is large

variation between each covariates examined (in the supplementary figures), a sensitivity analysis or

model comparison is highly recommended to statistically support the influence of these variables on

the model output. Comparing these models therefore can provide confident when reasoning whether

the covariables are necessary in the study.

**Authors:** We thank you, as well as reviewer 2, for encouraging us to explore the influence of additional covariates more thoroughly. We have formulated alternative models that include the influence of the ambient fluid medium (i.e. aquatic vs terrestrial realms) on the heat-dissipation time constants within both the saturating and hump-shaped model formulations. We have updated our supplementary results to include an extended LOOIC model comparison table (S1 Table) and included two additional tables (S2 and S3 Tables) which show the posterior parameter estimates from both these saturating and hump-shaped model formulations whose predictive performance we could not distinguish from the best-forming model based on LOOIC alone. However, these models both produce unrealistic parameter estimates for the heat-dissipation time constant implying that thermal conductance is greater in air than in water; we therefore reject these models in favour of the most parsimonious hump-shaped model, the *allometric heat-dissipation model* which includes a universal heat-dissipation time constant (i.e. animals experience a similar thermal conductance, irrespective of whether they move in air or water). We refrained from reporting the parameter estimates of the less parsimonious hump-shaped model (with variable slope) which also appears among the four best-performing models (see S1 Table). Our updated results section in the main text now also refers to these supplementary tables as well as the corresponding supplementary figures (S3 and S4 Figs). We appreciate this important addition that has helped to strengthen the take-home message of our manuscript. Please also see our reply to reviewer 2 on why we did not include thermoregulation in our model. We also added a short discussion on that topic to the revised manuscript (L340-359 and L388-393).

Methods:

To ensure standardised practice and transparency in reporting methods and results for comparative studies (Parker et al. 2018), I have included a “Transparency, openness, and reproducibility” checklist at the end of the document from the Tools for Transparency in Ecology and Evolution (TTEE; <https://osf.io/g65cb/>) website. This is to warrant appropriate methodological details reported in the study.

Parker, T. H., Griffith, S. C., Bronstein, J. L., Fidler, F., Foster, S., Fraser, H., Forstmeier, W., Gurevitch, J., Koricheva, J. and Seppelt, R. (2018). Empowering peer reviewers with a checklist to improve transparency. Nature Ecology & Evolution 2, 929-936.

**Authors:** We are grateful for providing us with the attached checklist and for making us aware of more recent literature that provides guidelines for improving the transparency and reproducibility of our study. We have updated the attached checklist.

The methods section was straight forward and easy to follow. There were some important information

missing from the data synthesis and analysis stage of the main document that requires further

clarification:

- What was the total records from the Web of Science and Google Scholar search?

- How many studies were retained and excluded?

- What was the inclusion and exclusion criteria for keeping studies in the data.

- Provide the proper references in the raw data instead of numbers.

- Provide the R code for the analysis,

- Provide references for the R packages used and the R software in the main document.

**Authors:** We have followed this suggestion and updated the methods section of our manuscript to include all of these points (L453-463) as well as the references for the R packages and software (lines). Our dataset, the corresponding reference list file, and the R code to reproduce the analysis have all been uploaded to Zenodo. DOI: 10.5281/zenodo.7554842

Stan Development Team. RStan: the R interface to Stan; 2022. R package version 2.21.5. Available from: http://­mc-stan.org/­.

R Core Team. R: A Language and Environment for Statistical Computing. Vienna, Austria; 2020. Available from: http://­www.R-project.org/­.

Vehtari A, Gabry J, Magnusson M, Yao Y, Bürkner PC, Paananen T, et al.. loo: Efficient leave-one-out cross-validation and WAIC for Bayesian models; 2022. R package version 2.5.0. Available from: https://­mc-stan.org/­loo/­.

Some additional caveats need to be acknowledged with the heat dissipation models, the model

assumes the animal is constantly moving for the heat-dissipation model to function and ignores how

many times the animal would need to rest. Under a hypothetical scenario it is fine but needs to be

stated.

**Authors:** It is correct that our concept of the time budget applies to an animal that is engaged in a state of sustained locomotion (e.g. exploration, dispersal, migration). We have now emphasised this in the caption for the conceptual figure (Fig 1). The caption now also emphasises the ‘cyclical’ manner in which heat-dissipation time is allocated (i.e. between each stride) (L128-129). Our first paragraph of the model development section introduces the concept by stating that ‘animals do not need to stop to dissipate heat; instead, they continuously allocate part of their total time budget towards heat-dissipation by moving more slowly’(L107-112). We hope that, when read together with the caption to Figure 1, this paragraph will be sufficient to clearly illustrate the concept to a general audience.

Tables

Table 1. Some missing descriptions of symbols such as P0 and E0, which I assume are the intercepts

of the power function?

**Authors:** Yes these are the intercepts to the power functions. We have now included these in the description of model parameters (L155).

Figures

Fig 1: For part C - time budget, is the fraction of heat-dissipation relative to body size or total?

**Authors:** We have updated our figure caption to clarify that the graphical representation of the total time budget of a moving animal is ‘relative to body mass’ (L132).

S1_Text

LS34-36: A recent study suggest metabolic-size scaling is due to evolutionary optimisation for life

history (White et al. 2022), while another theory suggests the scaling relationship is due to the

metabolic boundaries of cells (Glazier 2005, 2010). It would be worth considering when describing

mechanistic basis for metabolic-size scaling relationships.

Glazier, D. S. (2005). Beyond the ‘3/4-power law’: variation in the intra-and interspecific scaling of metabolic rate in animals. Biological Reviews, 80, 611-662.

Glazier, D. S. (2010). A unifying explanation for diverse metabolic scaling in animals and plants. Biological Reviews, 85, 111-138.

White, C. R., Alton, L. A., Bywater, C. L., Lombardi, E. J., & Marshall, D. J. (2022). Metabolic scaling is the product of life-history optimization. Science, 377, 834-839.

**Authors:** We thank you for highlighting the recent study by White et al (2022). We have now included it as an example of a more ‘holistic’ mechanistic hypothesis (LS37). We used the earlier review (Glazier 2005), which originally introduced the ‘metabolic-level boundaries hypothesis’, to justify a mechanism for the lower boundary constraint of 0.67 (LS33). We have now updated this paragraph by also citing Glazier (2005) to justify the upper bound for exercise-induced metabolic rate in relation to the metabolic boundaries of cells (LS35), while retaining the more specific studies related to the isometric scaling of muscle mitochondrial volume and muscle mass.

Eq 7: Is T in Celsius or Kevin?

**Authors:** Corrected: Both body temperature and the temperature of the ambient environment are specified by their SI units (Kelvin). (LS111)

Minor comments

L637: Journal missing in this paper.

**Authors:** Corrected

Transparency openness, and reproducibility checklist... [see attachment]

**Authors:** Thank you for your expertise in this matter and have attached the updated checklist.

**Reviewer #2:**

[identifies himself as Douglas S. Glazier]

General comments:

This is a fascinating, thoughtful and well-written study that attempts to explain why sustained speed during dispersal events relates to body mass in a humped (concave downward) left-skewed fashion. Previous publications have reported how and why maximum speed shows a humped relationship (e.g. Hirt et al. 2017), but this is the first study to show how and why this also occurs for sustained speed. I especially liked how the authors developed three alternative mathematical models and then compared them to the empirical data. Bravo! However, I have three major concerns that I believe that the authors should address.

**Authors:** Thank you for this concise summary of our paper and we are pleased to hear that our theory and approach was well received.

1) The authors consider 'conduction' in their heat-dissipation models, but apparently ignore 'convection' resulting from air/water flow. Therefore, their heat-dissipation models do not consider that an increased rate of body movement (speed) should increase heat dissipation via increased convection resulting from increased air/water movement across body surfaces. Therefore, there could be an interaction between speed and heat dissipation [i.e. between the mass scaling exponents for potential dispersal time (c) and heat-dissipation time (d)] that, if included, would change the predictions of their heat-dissipation models (i.e. reduce their downward curvature). This should be explored.

**Authors:** Thank you for this very insightful comment and for highlighting an important, but necessary, simplifying assumption which is implied by our application of Newton’s law of cooling. In line with previous work in the field of thermal biology (see McNab 1980), we consider overall thermal conductance, C (J s-1 K-1), as a ‘lumped’ parameter by assuming that both conductive and convective heat exchanges are approximately linearly related to the temperature differential between an animal’s core and its ambient environment. It is correct that this simplifying assumption does not consider how increased flow of air or water experienced by moving animals would lead to an increase in their overall thermal conductance via the increase in ‘forced’ convective heat exchange at the animal’s surface. We have refrained from modelling convective heat exchange as a function of travel speed as this would imply a feedback loop between travel speed to increased convective heat exchange, and, in turn, higher speed. The complexity of such a loop makes it difficult to include within an analytical model and would require simulations which would, in turn, also need to consider additional characteristics of the animal (e.g. its characteristic length, shape, and surface texture) as well as information on the ambient wind speed. Nevertheless, we agree that increased convection at higher travel speed should contribute to a reduced downward curvature of our allometric heat-dissipation model. We think that this may partially explain why we recovered a fitted value of 0.24 (90% CI: 0.19-0.29) for our allometric scaling exponent d rather than the value of 0.37 which we would have expected based on theoretical and empirical estimates of the scaling of maximal thermal conductance. We must also emphasise, however, that traits or behaviours that facilitate heat exchange via mass-independent pathways (the hypothesis described by our *constant heat-dissipation model*) would also contribute towards reducing the downward curvature of our model below the value of 0.37 (expected from a purely *allometric heat-dissipation model*) towards 0.0 (expected from a purely *constant heat-dissipation model*). Our results therefore suggest that the downward curvature in travel speed is explained by a blend of (primarily) mass-dependent and (to a lesser degree) mass-independent heat-dissipation constraints. This is well illustrated by whales who, despite being constrained by their large thermal inertia also employ behavioural (e.g. inhabiting icy polar waters) and physiological adaptations (e.g. counter-current vascular heat exchange in their fins) to increase heat loss (e.g. Scholander & Schevill 1950). We anticipate that extensions of our model that more accurately model convective heat loss by air or water flow around the body during motion as well as the characteristics of an animal’s shell/pelage (e.g. relative thickness, specific heat capacity) would help to disentangle the relative contributions of these extrinsic vs intrinsic constraints towards overall heat-dissipation and lead to a better understanding of the causes of decreasing travel speeds among the largest species. We have modified our discussion to include this point (L340-359 and L388-393) as a reference for future work. We highly appreciate this important addition to our manuscript.

Scholander, P.F. and Schevill, W.E., 1955. Counter-current vascular heat exchange in the fins of whales. *Journal of Applied Physiology*, *8*(3), pp.279-282

McNab B.K. On Estimating Thermal Conductance in Endotherms. Physiological Zoology. 1980;53:145–156.

> 2) I am concerned that the data presented in Fig. 2 do not show clear hump-shaped patterns. They appear to be largely indistinguishable from saturation curves because no marked negative decrease is obvious at large body sizes (unlike that seen for maximum speed, as reported by Hirt et al. 2017). Perhaps including the effects of convection in their models would help explain this. Please clarify.

**Authors:** We agree that an initial visual inspection of the data suggests that travel speed saturates with body mass. We have included a figure for the reviewers with different dimensions to illustrate how a visual impression might be misleading. Instead, by comparing the fits of our alternative mechanistic models to the data (of extant species) and interpreting the values of the parameter estimates (especially the normalisation constants), we can conclude that the relationships for flying, running and swimming animals must be hump-shaped. This became clearer after we incorporated your suggestion to consider aquatic vs terrestrial realm as a covariate to reflect that heat-dissipation in water is higher than in air (i.e. we expect the heat-dissipation time constant to be about four times smaller in water). Surprisingly, among our four best-performing models both the saturating and hump-shaped model which included this assumption yielded parameter estimates which predict the opposite (Tables S2 and S3, respectively) - they both make the unrealistic prediction that the speeds of swimming animals saturate sooner than those of running animals and imply that aquatic animals require more (4-10x) time to dissipate the heat that they generate while moving a unit distance (L203-210). Consequently, we reject this model in favour of the more parsimonious hump-shaped model with a single heat-dissipation time constant. We appreciate this discussion because we agree that initial visual inspection of the data gives a different impression than the statistical analyses. The emphasis on this point in the revised version of the manuscript has supported our main conclusion.

3) In my opinion, the authors should further explore how thermoregulatory mode (endothermy vs. ectothermy) and macrohabitat (aquatic vs. terrestrial) would affect the body-mass scaling of speed (already partially discussed in L 244-252). If the author's allometric heat-dissipation model is correct, then sustained speed should peak at lower body masses in endotherms vs. ectotherms, because endotherms typically have higher body temperatures at rest, thus reducing the increase in body temperature that can be tolerated. In addition, sustained speed should peak at lower body masses in terrestrial vs. aquatic species, because of greater heat dissipation in water, which has a higher heat capacity than air. However, unexpectedly no large difference in the scaling patterns exist between aquatic and terrestrial species (as seen in Fig. 2), and the endotherm-ectotherm comparison is complicated by the inclusion of flying birds and aquatic mammals. It would be useful to tease out these various effects because they importantly bear on the validity of the authors' heat dissipation models. Please clarify.

**Authors:** Thank you for the stimulating comments and for proposing interesting alternative hypotheses and model predictions. We hope that our reply to the previous comment sufficiently addresses the inclusion of macrohabitat/ambient medium (aquatic vs terrestrial) as a covariate. We also considered how best to incorporate thermoregulation (endo- vs ecto- vs mesotherm) as a covariate, both with respect to the locomotion rate constant v0 (i.e. endotherms have higher metabolic rates, and therefore higher rates of metabolic heat production given muscles with similar mechanical efficiencies) and the heat-dissipation time constant k0or k (i.e. endotherms have higher body temperatures at rest, thus reducing the increase in body temperature that can be tolerated). In both cases, however, we encounter the problem of  ‘confounding by cluster’ (see Silk et al. 2020) which is related to Simpon’s paradox; the categorical predictor (thermoregulation) is strongly correlated with body mass, i.e. body mass isn’t represented over a sufficient range in each level of the categorical predictor (e.g. flying ectotherms, flying endotherms, etc.) to fit this relationship. We think that while differences in thermoregulatory strategy (i.e. the degree of metabolic control over core body temperature) are important while animals are in a resting state, “the addition of locomotory demands which, during maximal aerobic activity, account for more than 90% of total oxygen demand” and which scales almost isometrically with body mass (see S1 Text, LS38-43) leads to a high degree of physiological equivalency (in terms of heat production) while animals are in a state of sustained locomotion. The fact that our *allometric heat dissipation model* has a single heat-dissipation time constant k, rather than separate constants for air vs water, also suggests that thermal conductance is a trait of the animal rather than a characteristic of its ambient medium. We suspect this arises because species-specific thermal properties such as insulation primarily serve animals while they are in a resting state; because animals are unable to drastically alter their insulation before switching into sustained state of locomotion (e.g. by temporarily removing their insulation) their only remaining strategy is to move more slowly in order to avoid hyperthermia. We have adjusted our discussion accordingly to include this interesting topic (L340-359 and L388-393) and appreciate this important addition to our manuscript.

Silk MJ, Harrison XA, Hodgson DJ. 2020. Perils and pitfalls of mixed-effects regression models in biology. *PeerJ* 8:e9522

> Specific comments:

> L 200-203: In the text the authors say that Fig. 2 depicts "a single hump-shaped relationship", but actually three relationships (three curved lines) are shown for each of the three major modes of locomotion. Please clarify.

**Authors:** We have followed this suggestion by clarifying that Fig. 2 depicts the three relationships for running, swimming and flying animals predicted by the best fitting hump-shaped scaling model (L200-203)

**Reviewer #3:**

This revised manuscript seems sound. I have no substantial comments. The details of the modeling are beyond my ability to critique. I did come across one reference that should be included and checked for data:

Garland, T., Jr., and R. L. Albuquerque. 2017. Locomotion, energetics, performance, and behavior: a mammalian perspective on lizards, and vice versa. Integrative and Comparative Biology 57:252-266.

**Authors:**

Thank you for this positive comment and for the additional reference. It is encouraging to read that our verbal and visual descriptions of the underlying models came across clearly. We did encounter the meta-study of Garland & Albuquerque (2017) during our literature search, however, we chose to exclude almost all the references from this study as these specifically represent estimates of ‘maximal aerobic speed’ (i.e. the speed at which VO2 max is attained) that were derived from treadmill studies where animals were usually stimulated to keep running at a particular speed. Our approach “precluded the use of movement data obtained from treadmills, flight mills, swim tunnels, wind tunnels, as well as from animals who were stimulated to move by an observer”. We recognise that treadmill-derived estimates of aerobic speed are critical to deriving the relationship between metabolic rate and speed, and thus, the allometry of the minimum metabolic energy cost of locomotion (maximum locomotion efficiency); however, a central argument of our *allometric heat-dissipation model* is that the larger animals fail to sustain their maximum aerobic speed (i.e. the speed predicted by our *metabolic model*) due to the increase in heat-dissipation time that arises as a consequence of their increase in body mass (larger animals have greater thermal inertia). Consequently, we only included aerobic speed data that were derived from animals which were free to choose and maintain their desired speed within an unrestrained setting (e.g. Gleeson 1979, cited in Garland & Albuquerque 2017), presumably in response to a combination of energetic and thermoregulatory constraints.

Gleeson, T. T. 1979. Foraging and transport costs in the Galapagos marine Iguana, *Amblyrhynchus cristatus*. Physiological Zoology 52:549–557.
