# Supplementary material for: The travel speeds of large animals are limited by their heat-dissipation capacities
Source: PLoS Biol. 2023 Apr 18;21(4):e3001820. doi: 10.1371/journal.pbio.3001820 (PMC10112811; doi:10.1371/journal.pbio.3001820)
Supplement: Attachment — Submitted filename: DyerA_2023_ReponseToReviewers_Rev2.docx [file pbio.3001820.s010.docx]

**Editor:**

Thank you for your patience while we considered your revised manuscript "Heat dissipation drives the hump-shaped scaling of animal travel speed with body mass" for publication as a Short Reports at PLOS Biology. This revised version of your manuscript has been evaluated by the PLOS Biology editors, the Academic Editor and the original reviewers.

Based on the reviews, we are likely to accept this manuscript for publication, provided you satisfactorily address the remaining points raised by reviewer #1 and the following data and other policy-related requests:

**Authors:**

Following the editor’s suggestion, we have changed the manuscript title to "The travel speeds of large animals are limited by their heat-dissipation capacities" and updated our Zenodo repository and cover letter accordingly.

We have clearly added the location of the underlying data to the legends of all main and supplementary tables and figures by including the sentence, “The data underlying this Figure can be found in <https://zenodo.org/record/7554842>"

In line with PLOC policy, we have removed the mention of the reviewers from the Acknowledgements.

We thank Douglas S. Glazier, Nicholas C. Wu and an anonymous reviewer for providing insightful feedback on our manuscript and Roli Roberts for facilitating a constructive review process.

REVIEWERS' COMMENTS:

Reviewer #1:

[identifies himself as Nicholas C Wu]

The authors did an excellent job addressing the reviewer's comments. The inclusion of a detailed description of the searches and data extraction is valuable for understanding how the data was obtained and what was included. The discussion is also more intuitive in interpreting the results. I have no further comments on the paper.

**Authors:** Thank you for your constructive review of your manuscript.

There are some minor comments to consider below:

L50-51: The terms "allee-, rescue-, source-sink, and drainage effects" might be too jargon for none experts. I would recommend describing them or just omit these examples.

**Authors:** We have omitted the examples of spatial dynamics from the introduction (L50-51)

L84: "scored", past tense for consistency.

**Authors:** Corrected

L422-424: Expand on the phrase "Therefore, allometric locomotion models such as our allometric heat dissipation model could provide the modular "building blocks" of dynamic meta-community or meta food web models" as it was not mentioned anywhere previously in the main text.

**Authors:** We have cited the same three references that were cited in L50-51 of the introduction as examples.

SI_Text.docx

Eq. 7: Is the Newtonian rate constant of temperature change independent to the surface area in this case? Make it clear, as Newton's law of cooling is the rate of cooling per unit surface area

**Authors:** Yes, we chose to start by introducing the non-dimensionalised form of Newton’s law of cooling in which the rate constant of temperature change has the units: 1/second. This simplified form can be derived from the dimensionalised form that explicitly includes area and a heat transfer coefficient with units: J/s/m^2. We use equation 8 to show that the rate constant of temperature change (1/s) is equal to an animal’s total thermal conductance (J/s/K) divided by its total heat capacity (J/K). Thermal conductance (J/s/K) is equal to the product of an animal’s surface area (m^2) and the heat transfer coefficient (J/s/m^2). Thermal biologists prefer to use body mass, which can be easily measured, as a proxy for surface area-related processes. We did then consider surface area-dependent heat exchange by showing that an animal’s thermal conductance (units: J/s/K, which is proportional to k) could either be independent of body mass (saturating model assumptions) or scale with body mass (hump-shaped model assumptions).
