# Supplementary material for: The travel speeds of large animals are limited by their heat-dissipation capacities
Source: PLoS Biol. 2023 Apr 18;21(4):e3001820. doi: 10.1371/journal.pbio.3001820 (PMC10112811; doi:10.1371/journal.pbio.3001820)
Supplement: Peer review history

# Editor report: Decision Letter 0

**DOI:** 10.1371/journal.pbio.3001820.r001

**Authors:** Roland G Roberts

5 Sep 2022

Dear Dr Dyer, 

Thank you for submitting your manuscript entitled "Heat dissipation drives the hump-shaped scaling of animal dispersal speed with body mass" for consideration as a Research Article by PLOS Biology.

Your manuscript has now been evaluated by the PLOS Biology editorial staff, as well as by an academic editor with relevant expertise, and I'm writing to let you know that we would like to send your submission out for external peer review.

IMPORTANT: We think that your manuscript would be better considered as a Short Report. No re-formatting is required at this moment, but please select "Short Reports" as the article type when you upload your additional metadata (see next paragraph).

However, before we can send your manuscript to reviewers, we need you to complete your submission by providing the metadata that is required for full assessment. To this end, please login to Editorial Manager where you will find the paper in the 'Submissions Needing Revisions' folder on your homepage. Please click 'Revise Submission' from the Action Links and complete all additional questions in the submission questionnaire.

Once your full submission is complete, your paper will undergo a series of checks in preparation for peer review. After your manuscript has passed the checks it will be sent out for review. To provide the metadata for your submission, please Login to Editorial Manager (https://www.editorialmanager.com/pbiology) within two working days, i.e. by Sep 07 2022 11:59PM.

If your manuscript has been previously peer-reviewed at another journal, PLOS Biology is willing to work with those reviews in order to avoid re-starting the process. Submission of the previous reviews is entirely optional and our ability to use them effectively will depend on the willingness of the previous journal to confirm the content of the reports and share the reviewer identities. Please note that we reserve the right to invite additional reviewers if we consider that additional/independent reviewers are needed, although we aim to avoid this as far as possible. In our experience, working with previous reviews does save time. 

If you would like us to consider previous reviewer reports, please edit your cover letter to let us know and include the name of the journal where the work was previously considered and the manuscript ID it was given. In addition, please upload a response to the reviews as a 'Prior Peer Review' file type, which should include the reports in full and a point-by-point reply detailing how you have or plan to address the reviewers' concerns. 

During the process of completing your manuscript submission, you will be invited to opt-in to posting your pre-review manuscript as a bioRxiv preprint. Visit http://journals.plos.org/plosbiology/s/preprints for full details. If you consent to posting your current manuscript as a preprint, please upload a single Preprint PDF. 

Feel free to email us at plosbiology@plos.org if you have any queries relating to your submission.

Kind regards,

Roli Roberts

Roland Roberts, PhD

Senior Editor

PLOS Biology

rroberts@plos.org

# Peer review: Decision Letter 1

**DOI:** 10.1371/journal.pbio.3001820.r002

**Authors:** Roland G Roberts

25 Oct 2022

Dear Dr Dyer,

Thank you for your patience while your manuscript "Heat dissipation drives the hump-shaped scaling of animal dispersal speed with body mass" was peer-reviewed at PLOS Biology. It has now been evaluated by the PLOS Biology editors, an Academic Editor with relevant expertise, and by three independent reviewers. 

In light of the reviews, which you will find at the end of this email, we would like to invite you to revise the work to thoroughly address the reviewers' reports.

As you will see below, the reviewers are broadly positive, but each has a number of requests that should be addressed before further consideration. For example, reviewer #1 asks that you re-frame the study away from “dispersal” and towards movement more generally (when asked to cross-comment, one other reviewer agreed strongly with this point), requests a sensitivity or model comparison, asks that you improve transparency of reporting (s/he provides a checklist), and wants some methodological clarifications and some caveats. Reviewer #2 asks you to explore the effects of convection, questions the interpretation of the data shown in Fig 2, and suggests that you also explore other variables, namely thermoregulatory mode (endothermy vs. ectothermy) and macrohabitat (aquatic vs. terrestrial).

Given the extent of revision needed, we cannot make a decision about publication until we have seen the revised manuscript and your response to the reviewers' comments. Your revised manuscript is likely to be sent for further evaluation by all or a subset of the reviewers.

We expect to receive your revised manuscript within 3 months. Please email us (plosbiology@plos.org) if you have any questions or concerns, or would like to request an extension. 

At this stage, your manuscript remains formally under active consideration at our journal; please notify us by email if you do not intend to submit a revision so that we may withdraw it.

**IMPORTANT - SUBMITTING YOUR REVISION**

Your revisions should address the specific points made by each reviewer. Please submit the following files along with your revised manuscript:

1. A 'Response to Reviewers' file - this should detail your responses to the editorial requests, present a point-by-point response to all of the reviewers' comments, and indicate the changes made to the manuscript. 

*NOTE: In your point-by-point response to the reviewers, please provide the full context of each review. Do not selectively quote paragraphs or sentences to reply to. The entire set of reviewer comments should be present in full and each specific point should be responded to individually, point by point.

You should also cite any additional relevant literature that has been published since the original submission and mention any additional citations in your response. 

2. In addition to a clean copy of the manuscript, please also upload a 'track-changes' version of your manuscript that specifies the edits made. This should be uploaded as a "Revised Article with Changes Highlighted" file type. 

*Re-submission Checklist*

When you are ready to resubmit your revised manuscript, please refer to this re-submission checklist: https://plos.io/Biology_Checklist

To submit a revised version of your manuscript, please go to https://www.editorialmanager.com/pbiology/ and log in as an Author. Click the link labelled 'Submissions Needing Revision' where you will find your submission record. 

Please make sure to read the following important policies and guidelines while preparing your revision:

*Published Peer Review*

Please note while forming your response, if your article is accepted, you may have the opportunity to make the peer review history publicly available. The record will include editor decision letters (with reviews) and your responses to reviewer comments. If eligible, we will contact you to opt in or out. Please see here for more details:

https://blogs.plos.org/plos/2019/05/plos-journals-now-open-for-published-peer-review/

*PLOS Data Policy*

Please note that as a condition of publication PLOS' data policy (http://journals.plos.org/plosbiology/s/data-availability) requires that you make available all data used to draw the conclusions arrived at in your manuscript. If you have not already done so, you must include any data used in your manuscript either in appropriate repositories, within the body of the manuscript, or as supporting information (N.B. this includes any numerical values that were used to generate graphs, histograms etc.). For an example see here: http://www.plosbiology.org/article/info%3Adoi%2F10.1371%2Fjournal.pbio.1001908#s5

*Blot and Gel Data Policy*

We require the original, uncropped and minimally adjusted images supporting all blot and gel results reported in an article's figures or Supporting Information files. We will require these files before a manuscript can be accepted so please prepare them now, if you have not already uploaded them. Please carefully read our guidelines for how to prepare and upload this data: https://journals.plos.org/plosbiology/s/figures#loc-blot-and-gel-reporting-requirements

*Protocols deposition*

To enhance the reproducibility of your results, we recommend that if applicable you deposit your laboratory protocols in protocols.io, where a protocol can be assigned its own identifier (DOI) such that it can be cited independently in the future. Additionally, PLOS ONE offers an option for publishing peer-reviewed Lab Protocol articles, which describe protocols hosted on protocols.io. Read more information on sharing protocols at https://plos.org/protocols?utm_medium=editorial-email&utm_source=authorletters&utm_campaign=protocols

Thank you again for your submission to our journal. We hope that our editorial process has been constructive thus far, and we welcome your feedback at any time. Please don't hesitate to contact us if you have any questions or comments.

Sincerely,

Roli Roberts

Roland Roberts, PhD

Senior Editor

PLOS Biology

rroberts@plos.org

------------------------------------

REVIEWERS' COMMENTS:

Reviewer #1:

[IMPORTANT: See fully formatted review provided in the PDF]

Major comments

This study developed a generalised biophysical model that integrates physiology in terms of

metabolic heat dissipation with locomotor capacity to explain average speed across body size from a

wide range of taxa. This is an interesting paper and I really enjoyed reading the logic and reasoning

behind the model. The main conceptual critique is on the term “dispersal” used throughout the paper.

While dispersal is ecologically important to understand how animals respond to a rapidly changing

world, the general allometric model is looking at average movement (Table 1 step 1) and energy

expenditure of locomotion (Table 1 step 3 and 4). I would recommend not limiting study to discussing

dispersal because dispersal is a behavioural process or state of a one-way, long-distance movement

to a new environment that is determined by intrinsic and extrinsic factors (Bowler & Benton 2005;

Stevens et al. 2014; Wu & Seebacher 2022), which locomotor capacity (maximum speed,

musculoskeletal capacity) and energy expenditure (metabolic rate, aerobic capacity) are one of the

limiting factors for the rate of dispersal. In fact, the description of the data collection section indicates

average speed from “maintaining sustained and directed movements within an unrestrained setting”

was extracted which could indicate general activity or movement that can encompasses foraging,

exploration, seeking shelter or mate, dispersal, or migration. There is no indication that the speeds

extracted were specific to dispersal. Therefore, the study itself looks at how heat dissipation affects

movement or locomotor capacity and not solely on dispersal. I would reframe the study to focus on

locomotor capacity, with part of the argument that dispersal is an important component of locomotion.

Therefore, the introduction, methods, results, and discussion should reflect this.

Bowler, D. E., & Benton, T. G. (2005). Causes and consequences of animal dispersal strategies: relating individual

behaviour to spatial dynamics. Biological Reviews, 80, 205-225.

Stevens, V. M., Whitmee, S., Le Galliard, J. F., Clobert, J., Böhning‐Gaese, K., Bonte, D., ... & Baguette, M. (2014).

A comparative analysis of dispersal syndromes in terrestrial and semi‐terrestrial animals. Ecology Letters, 17, 1039-

1052.

Wu, N. C., & Seebacher, F. (2022). Physiology can predict animal activity, exploration, and dispersal.

Communications Biology, 5, 1-11.

Results

L244-252: I disagree with this paragraph that the model did not include the covariables because they

did not correspond to systematic deviations. This was a visual assessment and while there is large

variation between each covariates examined (in the supplementary figures), a sensitivity analysis or

model comparison is highly recommended to statistically support the influence of these variables on

the model output. Comparing these models therefore can provide confident when reasoning whether

the covariables are necessary in the study.

Methods

To ensure standardised practice and transparency in reporting methods and results for comparative

studies (Parker et al. 2018), I have included a “Transparency, openness, and reproducibility” checklist

at the end of the document from the Tools for Transparency in Ecology and Evolution (TTEE;

https://osf.io/g65cb/) website. This is to warrant appropriate methodological details reported in the

study.

Parker, T. H., Griffith, S. C., Bronstein, J. L., Fidler, F., Foster, S., Fraser, H., Forstmeier, W., Gurevitch, J.,

Koricheva, J. and Seppelt, R. (2018). Empowering peer reviewers with a checklist to improve transparency. Nature

Ecology & Evolution 2, 929-936.

The methods section was straight forward and easy to follow. There were some important information

missing from the data synthesis and analysis stage of the main document that requires further

clarification:

- What was the total records from the Web of Science and Google Scholar search?

- How many studies were retained and excluded?

- What was the inclusion and exclusion criteria for keeping studies in the data.

- Provide the proper references in the raw data instead of numbers.

- Provide the R code for the analysis,

- Provide references for the R packages used and the R software in the main document.

Some additional caveats need to be acknowledged with the heat dissipation models, the model

assumes the animal is constantly moving for the heat-dissipation model to function and ignores how

many times the animal would need to rest. Under a hypothetical scenario it is fine but needs to be

stated.

Tables

Table 1. Some missing descriptions of symbols such as �! and �!, which I assume are the intercepts

of the power function?

Figures

Fig 1: For part C - time budget, is the fraction of heat-dissipation relative to body size or total?

S1_Text

LS34-36: A recent study suggest metabolic-size scaling is due to evolutionary optimisation for life

history (White et al. 2022), while another theory suggests the scaling relationship is due to the

metabolic boundaries of cells (Glazier 2005, 2010). It would be worth considering when describing

mechanistic basis for metabolic-size scaling relationships.

Glazier, D. S. (2005). Beyond the ‘3/4-power law’: variation in the intra-and interspecific scaling of metabolic rate in

animals. Biological Reviews, 80, 611-662.

Glazier, D. S. (2010). A unifying explanation for diverse metabolic scaling in animals and plants. Biological Reviews,

85, 111-138.

White, C. R., Alton, L. A., Bywater, C. L., Lombardi, E. J., & Marshall, D. J. (2022). Metabolic scaling is the product

of life-history optimization. Science, 377, 834-839.

Eq 7: Is T in Celsius or Kevin?

Minor comments

L637: Journal missing in this paper.

Transparency openness, and reproducibility checklist... [see attachment]

Reviewer #2:

[identifies himself as Douglas S. Glazier]

General comments:

This is a fascinating, thoughtful and well-written study that attempts to explain why sustained speed during dispersal events relates to body mass in a humped (concave downward) left-skewed fashion. Previous publications have reported how and why maximum speed shows a humped relationship (e.g. Hirt et al. 2017), but this is the first study to show how and why this also occurs for sustained speed. I especially liked how the authors developed three alternative mathematical models and then compared them to the empirical data. Bravo! However, I have three major concerns that I believe that the authors should address. 

1) The authors consider 'conduction' in their heat-dissipation models, but apparently ignore 'convection' resulting from air/water flow. Therefore, their heat-dissipation models do not consider that an increased rate of body movement (speed) should increase heat dissipation via increased convection resulting from increased air/water movement across body surfaces. Therefore, there could be an interaction between speed and heat dissipation [i.e. between the mass scaling exponents for potential dispersal time (c) and heat-dissipation time (d)] that, if included, would change the predictions of their heat-dissipation models (i.e. reduce their downward curvature). This should be explored. 

2) I am concerned that the data presented in Fig. 2 do not show clear hump-shaped patterns. They appear to be largely indistinguishable from saturation curves because no marked negative decrease is obvious at large body sizes (unlike that seen for maximum speed, as reported by Hirt et al. 2017). Perhaps including the effects of convection in their models would help explain this. Please clarify.

3) In my opinion, the authors should further explore how thermoregulatory mode (endothermy vs. ectothermy) and macrohabitat (aquatic vs. terrestrial) would affect the body-mass scaling of speed (already partially discussed in L 244-252). If the author's allometric heat-dissipation model is correct, then sustained speed should peak at lower body masses in endotherms vs. ectotherms, because endotherms typically have higher body temperatures at rest, thus reducing the increase in body temperature that can be tolerated. In addition, sustained speed should peak at lower body masses in terrestrial vs. aquatic species, because of greater heat dissipation in water, which has a higher heat capacity than air. However, unexpectedly no large difference in the scaling patterns exist between aquatic and terrestrial species (as seen in Fig. 2), and the endotherm-ectotherm comparison is complicated by the inclusion of flying birds and aquatic mammals. It would be useful to tease out these various effects because they importantly bear on the validity of the authors' heat dissipation models. Please clarify. 

Specific comments:

L 200-203: In the text the authors say that Fig. 2 depicts "a single hump-shaped relationship", but actually three relationships (three curved lines) are shown for each of the three major modes of locomotion. Please clarify.

Reviewer #3:

This revised manuscript seems sound. I have no substantial comments. The details of the modeling are beyond my ability to critique. I did come across one reference that should be included and checked for data:

Garland, T., Jr., and R. L. Albuquerque. 2017. Locomotion, energetics, performance, and behavior: a mammalian perspective on lizards, and vice versa. Integrative and Comparative Biology 57:252-266.

Attachment Submitted filename: PBIOLOGY-D-22-01874_R1 - Review.pdf Click here for additional data file.

# Author comment: Author response to Decision Letter 1

**DOI:** 10.1371/journal.pbio.3001820.r003

27 Jan 2023

Attachment Submitted filename: DyerA_2023_ReponseToReviewers_Rev1.docx Click here for additional data file.

# Peer review: Decision Letter 2

**DOI:** 10.1371/journal.pbio.3001820.r004

**Authors:** Roland G Roberts

20 Feb 2023

Dear Dr Dyer,

Thank you for your patience while we considered your revised manuscript "Heat dissipation drives the hump-shaped scaling of animal travel speed with body mass" for publication as a Short Reports at PLOS Biology. This revised version of your manuscript has been evaluated by the PLOS Biology editors, the Academic Editor and the original reviewers.

Based on the reviews, we are likely to accept this manuscript for publication, provided you satisfactorily address the remaining points raised by reviewer #1 and the following data and other policy-related requests:

IMPORTANT: Please address the following:

a) Please change your title to something more explicit and appealing. We suggest: "The travel speeds of large animals are limited by their heat-dissipation capacities."

b) Please attend to the remaining points raised by reviewer #1.

c) Thank you for provided all the underlying data and code in Zenodo. Please cite the location of the data clearly in all relevant main and supplementary Figure legends, e.g. “The data underlying this Figure can be found in https://zenodo.org/record/7554842"

d) I note that you mention the reviewers in the Acknowledgements. While we appreciate the sentiment, this is against PLOS policy, so please could you remove this?

As you address these items, please take this last chance to review your reference list to ensure that it is complete and correct. If you have cited papers that have been retracted, please include the rationale for doing so in the manuscript text, or remove these references and replace them with relevant current references. Any changes to the reference list should be mentioned in the cover letter that accompanies your revised manuscript.

We expect to receive your revised manuscript within two weeks. 

To submit your revision, please go to https://www.editorialmanager.com/pbiology/ and log in as an Author. Click the link labelled 'Submissions Needing Revision' to find your submission record. Your revised submission must include the following:

- a cover letter that should detail your responses to any editorial requests, if applicable, and whether changes have been made to the reference list

- a Response to Reviewers file that provides a detailed response to the reviewers' comments (if applicable)

- a track-changes file indicating any changes that you have made to the manuscript. 

NOTE: If Supporting Information files are included with your article, note that these are not copyedited and will be published as they are submitted. Please ensure that these files are legible and of high quality (at least 300 dpi) in an easily accessible file format. For this reason, please be aware that any references listed in an SI file will not be indexed. For more information, see our Supporting Information guidelines:

https://journals.plos.org/plosbiology/s/supporting-information

*Published Peer Review History*

Please note that you may have the opportunity to make the peer review history publicly available. The record will include editor decision letters (with reviews) and your responses to reviewer comments. If eligible, we will contact you to opt in or out. Please see here for more details:

https://blogs.plos.org/plos/2019/05/plos-journals-now-open-for-published-peer-review/

*Press*

Should you, your institution's press office or the journal office choose to press release your paper, please ensure you have opted out of Early Article Posting on the submission form. We ask that you notify us as soon as possible if you or your institution is planning to press release the article.

*Protocols deposition*

To enhance the reproducibility of your results, we recommend that if applicable you deposit your laboratory protocols in protocols.io, where a protocol can be assigned its own identifier (DOI) such that it can be cited independently in the future. Additionally, PLOS ONE offers an option for publishing peer-reviewed Lab Protocol articles, which describe protocols hosted on protocols.io. Read more information on sharing protocols at https://plos.org/protocols?utm_medium=editorial-email&utm_source=authorletters&utm_campaign=protocols

Please do not hesitate to contact me should you have any questions.

Sincerely,

Roli Roberts

Roland Roberts, PhD

Senior Editor,

rroberts@plos.org,

PLOS Biology

------------------------------------------------------------------------

DATA NOT SHOWN?

- Please note that per journal policy, we do not allow the mention of "data not shown", "personal communication", "manuscript in preparation" or other references to data that is not publicly available or contained within this manuscript. Please either remove mention of these data or provide figures presenting the results and the data underlying the figure(s).

------------------------------------------------------------------------

REVIEWERS' COMMENTS:

Reviewer #1:

[identifies himself as Nicholas C Wu]

The authors did an excellent job addressing the reviewer's comments. The inclusion of a detailed description of the searches and data extraction is valuable for understanding how the data was obtained and what was included. The discussion is also more intuitive in interpreting the results. I have no further comments on the paper.

There are some minor comments to consider below:

L50-51: The terms "allee-, rescue-, source-sink, and drainage effects" might be too jargon for none experts. I would recommend describing them or just omit these examples.

L84: "scored", past tense for consistency.

L422-424: Expand on the phrase "Therefore, allometric locomotion models such as our allometric heat dissipation model could provide the modular "building blocks" of dynamic meta-community or meta food web models" as it was not mentioned anywhere previously in the main text.

SI_Text.docx

Eq. 7: Is the Newtonian rate constant of temperature change independent to the surface area in this case? Make it clear, as Newton's law of cooling is the rate of cooling per unit surface area

Reviewer #2:

[identifies himself as Douglas S. Glazier]

Good job!

# Author comment: Author response to Decision Letter 2

**DOI:** 10.1371/journal.pbio.3001820.r005

6 Mar 2023

Attachment Submitted filename: DyerA_2023_ReponseToReviewers_Rev2.docx Click here for additional data file.

# Editor report: Decision Letter 3

**DOI:** 10.1371/journal.pbio.3001820.r006

**Authors:** Roland G Roberts

7 Mar 2023

Dear Dr Dyer,

Thank you for the submission of your revised Short Report "The travel speeds of large animals are limited by their heat-dissipation capacities" for publication in PLOS Biology. On behalf of my colleagues and the Academic Editor, Anders Hedenström, I'm pleased to say that we can in principle accept your manuscript for publication, provided you address any remaining formatting and reporting issues. These will be detailed in an email you should receive within 2-3 business days from our colleagues in the journal operations team; no action is required from you until then. Please note that we will not be able to formally accept your manuscript and schedule it for publication until you have completed any requested changes.

Please take a minute to log into Editorial Manager at http://www.editorialmanager.com/pbiology/, click the "Update My Information" link at the top of the page, and update your user information to ensure an efficient production process.

PRESS: We frequently collaborate with press offices. If your institution or institutions have a press office, please notify them about your upcoming paper at this point, to enable them to help maximise its impact. If the press office is planning to promote your findings, we would be grateful if they could coordinate with biologypress@plos.org. If you have previously opted in to the early version process, we ask that you notify us immediately of any press plans so that we may opt out on your behalf.

We also ask that you take this opportunity to read our Embargo Policy regarding the discussion, promotion and media coverage of work that is yet to be published by PLOS. As your manuscript is not yet published, it is bound by the conditions of our Embargo Policy. Please be aware that this policy is in place both to ensure that any press coverage of your article is fully substantiated and to provide a direct link between such coverage and the published work. For full details of our Embargo Policy, please visit http://www.plos.org/about/media-inquiries/embargo-policy/.

Thank you again for choosing PLOS Biology for publication and supporting Open Access publishing. We look forward to publishing your study. 

Sincerely, 

Roli Roberts

Roland G Roberts, PhD, PhD

Senior Editor

PLOS Biology

rroberts@plos.org
